# Intermittent fasting enhances long-term memory consolidation, adult hippocampal neurogenesis, and expression of longevity gene Klotho

**DOI:** 10.1038/s41380-021-01102-4

**Published:** 2021-05-25

**Authors:** Gisele Pereira Dias, Tytus Murphy, Doris Stangl, Selda Ahmet, Benjamin Morisse, Alina Nix, Lindsey J. Aimone, James B. Aimone, Makoto Kuro-O, Fred H. Gage, Sandrine Thuret

**Affiliations:** 1 Department of Basic and Clinical Neuroscience, Institute of Psychiatry, Psychology and Neuroscience, King’s College London, London, UK; 2 Laboratory of Genetics, The Salk Institute for Biological Studies, La Jolla, CA USA; 3 Center for Computing Research, Sandia National Laboratories, Albuquerque, NM USA; 4 Division of Anti-Ageing Medicine, Center for Molecular Medicine, Jichi Medical University, Tochigi, Japan; 5 Department of Neurology, University Hospital Carl Gustav Carus, Technische Universität Dresden, Dresden, Germany

**Keywords:** Stem cells, Neuroscience

## Abstract

Daily calorie restriction (CR) and intermittent fasting (IF) enhance longevity and cognition but the effects and mechanisms that differentiate these two paradigms are unknown. We examined whether IF in the form of every-other-day feeding enhances cognition and adult hippocampal neurogenesis (AHN) when compared to a matched 10% daily CR intake and ad libitum conditions. After 3 months under IF, female C57BL6 mice exhibited improved long-term memory retention. IF increased the number of BrdU-labeled cells and neuroblasts in the hippocampus, and microarray analysis revealed that the longevity gene *Klotho* (Kl) was upregulated in the hippocampus by IF only. Furthermore, we found that downregulating Kl in human hippocampal progenitor cells led to decreased neurogenesis, whereas Kl overexpression increased neurogenesis. Finally, histological analysis of Kl knockout mice brains revealed that Kl is required for AHN, particularly in the dorsal hippocampus. These data suggest that IF is superior to 10% CR in enhancing memory and identifies Kl as a novel candidate molecule that regulates the effects of IF on cognition likely via AHN enhancement.

## Introduction

Calorie restriction (CR), typically defined as a 10–40% total reduction in daily calorie intake, and intermittent fasting (IF), typically involving every-other-day feeding, are two established dietary paradigms that extend life- and health-span across species [1–5]. A recent study demonstrated that adoption of 30% CR or a single meal feeding strategy for 10 months enhanced longevity and health status in mice, regardless of whether their content was high or low in sugar [6]. Adherence to CR and IF regimens also improves learning and memory in different models [7–11]. Mechanistically, animal studies have shown that CR and IF induce a mild adaptive cellular stress response that promotes neuronal resilience to injury and pathology [12]. Notably, CR and IF have been conflated in the literature with many studies reporting the beneficial effects of CR on measures of inflammation [13], neurodegeneration [14], brain plasticity [15, 16], learning and motor performance [17], when in fact forms of IF were used in these studies to bring about overall reductions in calorie intake. However, one of the first studies to dissociate IF from CR demonstrated that mice in the IF paradigm did not reduce their overall food intake and maintained body weight [18]. Despite no reduction in total calorie intake, IF still induced beneficial effects that matched those elicited by 40% CR, including reduced concentrations of serum insulin and glucose when compared to ad libitum (AL) intake. IF also improved neuronal survival following excitotoxic challenge in hippocampal regions CA1 and CA3 as induced by a stereotaxic injection of kainate in the dorsal hippocampus (DH) when compared to CR and AL mice [18]. It is important to acknowledge, however, that the similar body weight between IF and CR groups was only achieved 20 weeks after the start of the dietary regimens. It is possible that effects on brain plasticity driven by short-term differences in overall body weight between groups may still be a factor that hinders the disentanglement of cellular and molecular pathways that are specific to IF and CR. Even with these potential limitations, the findings by Anson et al. [18] indicate that IF could induce neuroprotection independent of overall calorie intake in the long term.

Despite the positive effects of CR and IF in neurodegenerative [19, 20] and affective [21, 22] conditions, the specific behavioral contributions and mechanisms that differentiate both interventions remain largely unknown. Answering these questions is pivotal to adapting these regimens to human populations, given the challenges of adhering to a long-term CR regimen when compared to the improved adherence to variations of the IF paradigm [23]. Here, we directly compared the effects of IF to a matched 10% daily CR regimen upon learning and memory in mice. A 10% energy restriction protocol was chosen for the CR group following the observation that IF mice overall consume 10% less calories on a weekly basis. IF improved long-term retention memory to a greater extent than CR and was associated with increased adult hippocampal neurogenesis (AHN) and upregulation of the longevity gene *Klotho* (Kl). The Kl gene produces a membrane-bound, single-pass protein (KL) that can be cleaved at the cell surface to produce a secreted form found in mammalian sera, urine and cerebrospinal fluid [24–30]. Though KL is produced primarily in the kidney, it is also highly expressed in some brain areas, including the dentate gyrus (DG) of the hippocampus and in particular by its mature neurons [31]. The function of Kl in the brain is still largely unknown but it has been proposed that Kl plays an important role in cognition because increased serum levels of KL were associated with increased cognitive ability in humans and rodents [25]. Here, we confirm previous evidence suggesting that Kl is an important regulator of AHN [32, 33] and propose it as a novel molecular player through which IF may enhance cognitive performance. Finally, we highlight the potential for IF paradigms in the form of increased meal interval to help bring about improved cognitive performance in human populations.

## Materials and methods

For a detailed description of the materials and methods, please refer to the Supplementary information.

### Animals

Seventy-five 8-week-old female C57BL6 mice were assigned into three groups: 25 AL fed control, 25 CR (10% less than AL), and 25 IF (every-other-day feeding, totaling in a 10% weekly reduction in calorie consumption). The mice were subjected to these regimens for 3 months, after which three per group were used for gene expression studies to investigate the molecular mechanisms involved in AHN (Supplementary Fig. 1). The remaining mice were used for behavioral studies (Morris water maze (MWM); *n* = 5 AL, 10 CR, and 10 IF) and were injected with bromodeoxyuridine (BrdU) for histology (*n* = 12/group). Half of the BrdU-injected mice were sacrificed 24 h after the last injection; the remaining mice were sacrificed 4 weeks after the last BrdU injection (Supplementary Fig. 1). All animal procedures were in accordance with NIH guidelines and approved by the Salk Institutional Animal Care and Use Committee.

For the histological analyses of hippocampal neurogenesis in *kl/kl* mice, brains from 8-week-old male (*n* = 8 Wt; 5 *kl/kl*) and female (*n* = 7 Wt; 8 *kl/kl*) rodents were used.

### Morris water maze (MWM)

Procedures for the assessment of spatial learning and memory in the AL, CR, and IF animals followed those previously described [34].

### BrdU administration

Dietary intervention experiments: animals (*n* = 12 AL; 12 CR; 12 IF) received a daily intraperitoneal BrdU (50 µg/g body weight) injection for 6 consecutive days, and euthanasia followed either 24 h (*n* = 6 mice/group) or 4 weeks (*n* = 6 mice/group) after the last BrdU injection.

Kl mice: animals (*n* = 5 Wt [3 males, 2 females]; 5 *kl/kl* [2 males, 3 females]) received three intraperitoneal injections of BrdU (100 mg/kg) administered at 10 mg/ml, approximately every 16 h, and euthanasia followed 4 weeks after the last BrdU injection.

### Euthanasia and brain sectioning

Animals were anesthetized with ketamine (100 mg/kg) and xylazine (10 mg/kg) and perfused through the left ventricle of the heart with saline and 4% paraformaldehyde in 0.1 M phosphate buffer. Brains were removed, immersed in 4% PA solution for 24 h, and cryoprotected in 30% sucrose in 0.1 M phosphate buffer at 4 °C. Brain sections (40 μm) were obtained using a microtome apparatus (Microm, Germany). Sections containing the DG of the hippocampus were collected in a 96-well plate and stored at −20 °C. Sections were stored in tissue cryo-protective solution.

### Immunostaining of free-floating sections and immunofluorescence

For each diet group or Kl brain, one-in-six series of sections were transferred into one well of a six-well plate. Staining procedures followed those previously described [34]. Analysis of the septo-temporal distribution of doublecortin^+^ (DCX^+^) cells in the DG of diet mice, as well as a dendrite quantitative morphometric analysis, was also undertaken, following the immunostaining of sections for DCX (Supplementary Fig. 2A, B). Additional details can be found in the Supplementary information (Supplementary Table 1).

### Threshold analysis of KL

Genome-wide expression analysis was used to investigate genes upregulated by IF. Differentially expressed genes between IF and CR were also taken forward for pathway analysis using DAVID (https://david.ncifcrf.gov/) and ingenuity pathway analysis (IPA, http://www.ingenuity.com, Qiagen, USA) (Supplementary Fig. 3). Validation of the array by RT-PCR was also undertaken, confirming upregulation of K1 under IF (Supplementary Fig. 4 and Supplementary Table 2). Furthermore, expression of Kl in the mouse brain indicated a potential role for this gene in the hippocampus (Supplementary Fig. 5). To confirm this finding in our diet brains, ten non-overlapping images of AL, CR, and IF sections immunostained for KL were captured from three consecutive sections per animal (*n* = 3/group). Images were obtained with a live video camera (JVC, 3CCD, KY-F55B) mounted onto a Zeiss Axioplan microscope using a 5× objective. Parameters including lamp intensity, video camera setup, and calibration were kept constant throughout the analysis. Subsequently images were analyzed using the Image Pro Plus 4.0 (Media Cybernetics) image analysis software. An appropriate threshold that selected foreground immunoreactivity above background was applied as a constant for all images analyzed.

### Stereological and confocal analysis

The optical fractionator [35] method of unbiased stereology was used to count the number of DAB-revealed positive cells in sections of AL, CR, and IF brains (BrdU; DCX), as well as in *kl/kl* and Wt brains (DCX). Procedures for both stereology and confocal microscopy followed those previously described [34]. The criteria used to differentiate the DH and ventral hippocampus (VH) followed those described previously [36]. Specifically, brain slices within −1.06 to −2.06 mm relative to bregma were used for the DH, and those containing coordinates −3.08 to −3.80 mm were used for the VH, in accordance with the Paxinos and Franklin’s brain atlas [37].

### In vitro assays

Cells from the human hippocampal neural progenitor cell line HPCOA07/03 (ReNeuron Ltd) were used as an in vitro model of human hippocampal neurogenesis [38–41] to investigate whether Kl plays a role in regulating different stages of neurogenesis. Procedures for growing the HPCOA07/03 cell line followed those previously described [38] and were tested monthly as free of mycoplasma.

### Generation of Kl overexpressing cell line (*Klover*)

To assess the effect of Kl on the proliferation and differentiation of HPCOA07/03 cells, these cells were genetically engineered to conditionally overexpress the secreted form of KL using the Lenti-XTM Tet-On^®^ Advanced Inducible Expression System (Clonetech). Gene expression is activated in this system using the tetracycline Doxycycline. The Lenti-XTM Tet-On^®^ Advanced Inducible Expression System consists of a regulator vector, pLVX-Tet-On Advanced, and a response vector, pLVX-Tight-Puro. The regulator vector constitutively expresses a tetracycline-controlled transactivator (rtTA-Advanced) that, in the presence of Doxycycline, binds to the inducible promoter (Ptight) of the gene of interest in the response vector and activates transcription. Ptight consists of a tet-responsive element joined to a minimal CMV promoter. Induction of the system produces high-level transcription of the gene of interest.

### Cloning of secreted Kl into the pVLX-Tight-Puro vector/sequencing of the Kl secreted pVLX-Tight-Puro plasmid

Please refer to the Supplementary information (Supplementary Table 3).

#### Lentivirus generation

For the transfection of the HPCOA07/03, the modified Lenti-TM Tet-On^®^ Advanced Inducible Expression System (Clonetech) was used. To generate and package this virus, the envelope plasmid pMDG:VSV-G and the packaging plasmid P8.91:GAG-POL were used instead of the Lenti-X HT packaging mix, and the transfection was conducted using Lipofectamine instead of Lentiphos HT, as this setup was already in place in the lab. To generate the two viruses, one with the regulator vector and one with the response vector, HEK cells were transfected using Lipofectamine following the manufacturer’s instructions, with a total of 30 μg of DNA containing the envelope plasmid pMDG:VSV-G, the packaging plasmid P8.91:GAG-POL, and either the regulator or the response plasmid. The virus was collected 24 and 48 h after the transfection and concentrated using the Lenti-X concentrator (Clontech) following the manufacturer’s instructions. The concentrated virus was stored in aliquots at −80 °C.

#### Transduction of the HPCOA07/03 cell line using lentivirus

HPCOA07/03 cells were cultured for 48 h as described previously; media was changed 24 h before the viral transduction. Cultures were transducted in six-well plates (Nunc) at 60% confluency. Five microliter of regulator virus and 10 μl of response virus were added to 2 ml of media and centrifuged for 45 min at 750 g, 32 °C. After 30-min incubation at 37 °C, the media was changed and 48 h later the cells were subjected to antibiotic selection using 0.2 μl/ml Puromycin. The Kl secreted pVLX-Tight-Puro expresses resistance to the antibiotic Puromycin. To ensure only successfully transducted cells were cultured, the cells were further subjected to Puromycin for 2 days. Cells not carrying the plasmid were dead after 2 days. The pLVX-Tet-On Advanced plasmid expresses resistance to the antibiotic Neomycin; however, the HPCOA07/03 cells are already resistant to Neomycin after they have been made conditionally immortalized with the c-myc-ER. Nonetheless, the successful transduction of both plasmids was confirmed by immunocytochemistry against the secreted form of KL. The protein of the gene of interest can only be expressed if the cell carries both plasmids, the regulator, and the response vector. Cells, further referred to as *Klover*, were then passed into a T75 flask (Nunc) and cultured or frozen as needed.

#### *Klover* assay

To induce expression of the secrete form of KL in the *Klover* HPCOA07/03 cells, cultures were treated with Doxycycline (Sigma) (1 μg/ml) 24 h after seeding for 24 h. For the proliferation assay, cells were cultured for 3 days under proliferation conditions; for the differentiation assay, cell differentiation was started at the same time as Kl expression was started by adding Doxycycline (Supplementary Fig. 6). *Klover* cells were also immunostained for several neurogenesis markers, as reported in the main results and Supplementary Fig. 7. Additional details can be found in the Supplementary information.

### Generation of Kl knocked-down cell line

#### RNA interference

To suppress the protein expression of KL, cells were transfected with stealth siRNA (Invitrogen) that specifically binds to Kl mRNA in the cytoplasm (Supplementary Table 4). HPCOA07/03 cells were transfected using the N-TER Nanoparticle siRNA Transfection System (5 nM) (Sigma N2913), and BLOCK-iT Alexa Fluor red Fluorescent Oligo (5 nM) (Invitrogen) was used to determine the efficiency of transfection, which was estimated at >80%. HPCOA07/03 cells were transfected 24 h after siRNA transfection and maintained under differentiating conditions for 7 days (Supplementary Fig. 8). siRNA studies used an *n* of 2 representing two independent experiments, each with three technical replicates (specifically, the experiments were performed on two independent passages done in three independent plates, totaling six independent technical replicates).

#### Immunocytochemistry

A range of antibodies (Supplementary Table 5) was used to assess the proliferation, differentiation, and survival of progenitor cells after they were differentiated for 3 or 7 days in *Klover* conditions or for 7 days in Kl knocked-down assays. Additional details can be found in the Supplementary information.

#### Cell microscopy

Pictures of immunocytochemically stained cells were taken with a Cell Insight high content imaging platform (Thermo Fisher Scientific, Ltd) and a fluorescence microscope Axio Imager microscope (Carl Zeiss Inc.) using the Axio vision Digital Image Processing Software Version 4.67.1 (Carl Zeiss Inc.). Bright field images were taken with the Olympus inverted microscope using the same imaging software. Pictures were imported into ImageJ (http://rsbweb.nih.gov/ij/) for further processing.

#### Data analysis

In the dietary intervention experiments, behavioral data were analyzed in GraphPad Prism 8 using Two-Way ANOVA with Tukey’s Multiple Comparison Test. Histological data and RT-QPCR of diet brains were analyzed in GraphPad Prism 8 using one-way ANOVA with Tukey’s multiple comparison test. Differences were considered significant when *p* ≤ 0.05. For in vitro experiments, in vitro data were analyzed in GraphPad Prism 8 using Student’s *t*-test for unpaired samples (expressed as mean ± SEM; *Klover* assays) or one-way ANOVA with Tukey’s post hoc test for multiple comparisons among treatment groups (siRNA studies; adjusted *p* values from group comparisons can be found in Supplementary Table 6). The number of biological replicates was three if not otherwise stated. Differences were considered significant when *p* ≤ 0.05. Data from *kl/kl* × Wt brains were analyzed in GraphPad Prism 8 using Student’s *t*-test for unpaired samples (expressed as mean ± SEM). Differences were considered significant when *p* ≤ 0.05.

## Results

### IF is more effective than CR in promoting long-term memory retention and increasing the number of neuroblasts in the DG

**Fig. 1:**
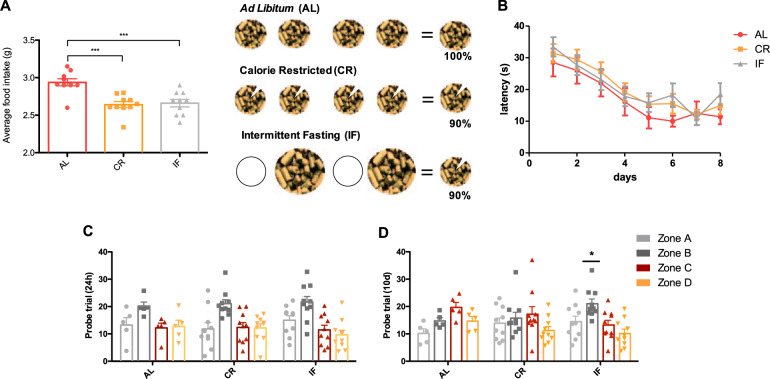
Spatial learning and memory in mice subjected to different dietary regimes: ad libitum (AL), 10% calorie restriction (CR), and intermittent fasting (IF). Female C57Bl6 mice on IF consume overall only 10% less than those fed ad libitum (**A**). To compare the effects of IF and CR on cognitive performance, while controlling for calorie intake, mice were subjected to one of three diet conditions for 3 months: AL, 10% CR, or IF (*n* = 20 AL, 25 CR, and 25 IF) (**A**). After an acquisition phase on the Morris water maze, no differences were found between groups regarding spatial learning (**B**). No differences were found in a probe trial test performed to assess memory retention at 24 h (**C**). However, in a probe trial test at 10 days after the last training trial in the acquisition phase, IF mice performed significantly better, spending 30 and 25% more time in the correct quadrant zone (zone B) when compared to both AL and CR animals, respectively (**D**). *p ≤ 0.05.

We observed that female C57Bl6 mice on an IF diet consumed overall only 10% less than those fed AL (Fig.1A). To directly compare the effects of IF and CR on cognitive performance, while controlling for calorie intake, female C57Bl6 mice were subjected to one of three diet conditions for 3 months: AL, 10% CR, or IF (*n* = 25/group) (Fig.1A).

The MWM was used to assess cognitive performance. After two consecutive trials per day from different quadrants in the acquisition phase, no differences were found between groups across the 8 days of testing (Fig. 1B). A probe trial test was performed to assess memory retention at 24 h (Fig. 1C) and 10 days (Fig. 1D) post training. No differences were detected between groups in the 24-h probe trial task (*F*(6, 88) = 0.57; *p* = 0.7528; AL, *n* = 5; CR, *n* = 10; IF, *n* = 10); in contrast, at 10 days after the last training trial in the acquisition phase, IF mice performed significantly better, spending 30 and 25% more time in the correct quadrant zone (zone B) when compared to both AL and CR animals, respectively (*F*(6, 88) = 2.424; *p* = 0.0325; AL, *n* = 5; CR, *n* = 10; IF, *n* = 10).

**Fig. 2:**
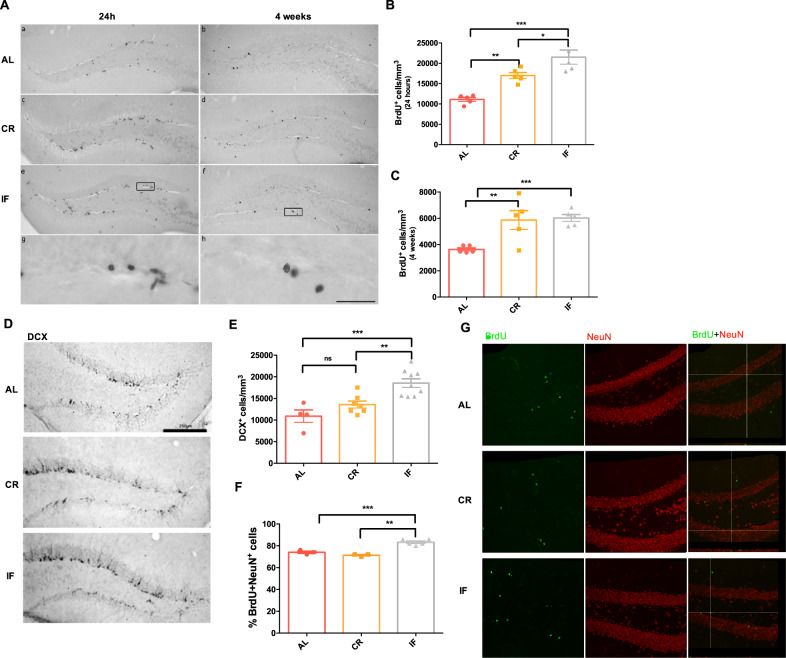
Cell proliferation, DCX expression, and neuronal survival in the dentate gyrus of C57BL/6 mice upon different diet paradigms. Photomicrographs of BrdU^+^ cells in the dentate gyrus 24 h after the last BrdU injection in (**A**: **a**, **c**, **e**, **g**) or 4 weeks after the last BrdU injection (**A**: **b**, **d**, **f**, **h**) in mice fed ad libitum (AL) (**A**: **a**, **b**), under calorie restriction (CR) conditions (**A**: **c**, **d**), or under intermittent fasting (IF) conditions (**A**: **e**, **f**). **g** and **h** are higher magnification photomicrographs of the boxed area in **e** and **f**, respectively. The number of BrdU^+^ cells per DG in CR and IF conditions was superior to AL 24 h (**B**) and 4 weeks (**C**) after BrdU injections. IF was also superior to CR regarding number of BrdU^+^cells per DG 24 h post injection (**B**). Photomicrographs of DCX^+^ cells in the dentate gyrus of AL, CR, and IF mice (**D**). The number of DCX^+^ neuroblasts was also increased in the IF group in comparison with both AL and CR, respectively (**E**). Analysis of co-labeled cells with BrdU and the nuclear neuronal marker NeuN revealed that IF mice significantly increased the proportion of co-labeled cells when compared to AL and CR animals, respectively (**F**). Scale bars, 100 μm (**a**–**f**), 35 μm (**g**, **h**), 250 μm (**D**). BrdU bromodeoxyuridine, DCX doublecortin, DG dentate gyrus, NeuN neuronal nuclei protein. *p ≤ 0.05; **p ≤ 0.01; ***p ≤ 0.001.

We next investigated if the differential effects of IF and CR on memory retention would be accompanied by specific cellular and molecular changes. Evidence for an association between increased AHN and improved spatial learning and memory retention [42–45] informed the choice for investigating neurogenic parameters in the present study. With respect to cell proliferation, as estimated by the total number of cells having incorporated BrdU 24 h post injection, there was a statistically significant difference between groups, with the number of cells in the AL and CR groups being significantly lower than that in the IF group (*F*(2,14) = 21.02, *p* = 0.0001; AL: 11,105 ± 494.4, *n* = 5; CR: 16,987 ± 762.4, *n* = 5; IF: 21,504 ± 1747, *n* = 5) (Fig. 2A, B). In the 4-week post BrdU-injected brains, we observed a significant increase in surviving adult-born cells in the CR and IF groups compared to the AL group (*F*(2,14) = 8.788, *p* = 0.0045; AL: 3627 ± 120.8, *n* = 5; CR: 5862 ± 724.6, *n* = 5; IF: 6022 ± 269.5, *n* = 5) (Fig. 2A, C). There were no differences in 4-week survival between the CR and IF groups. The number of DCX^+^ neuroblasts was also significantly increased in the IF group in comparison with both AL and CR (*F*(2,17) = 12.95, *p* = 0.0004; AL: 10,902 ± 1462, *n* = 4; CR: 13,581 ± 817.5, *n* = 7; IF: 18,542 ± 1005, *n* = 9) (Fig. 2D, E). Analysis of co-labeled cells with BrdU and the nuclear neuronal marker NeuN revealed that IF mice had a significant increase in the percentage of co-labeled cells when compared to AL and CR animals (*F*(2, 8) = 31.75, *p* = 0.0002; AL: 74 ± 1.155, *n* = 3; CR: 71.33 ± 0.667, *n* = 3; IF: 83.20 ± 1.200, *n* = 5), indicating an increase in neuronal differentiation following IF (Fig. 2F, G). No differences were observed between groups with regard to either total number of dendritic branches per cell or dendritic length (Supplementary Fig. 2B). These results indicate that IF increased cell proliferation in the DG and the generation of neuroblasts and survival of neurons to a greater extent than CR.

### Genome-wide expression analysis reveals Kl is upregulated by IF

A genome-wide microarray analysis of hippocampal gene expression revealed a distinct gene profile for IF mice when compared to both AL and CR (raw extended data on Figshare 10.6084/m9.figshare.14241842.v1). Seventy probes were differentially expressed in the IF vs. AL analysis (Supplementary Spreadsheet 1) and gene ontology analysis revealed enrichment for keywords related to “copper” and “ion transport” (Supplementary Spreadsheet 2). IPA also showed that differential IF vs. AL genes resulted in predicted activation states for increased “cognition” and decreased “organismal death” (Supplementary Spreadsheet 3). Upon comparing IF to CR, 186 probes were differentially expressed (Supplementary Spreadsheet 1 and Supplementary Fig. 3) and gene ontology analysis demonstrated a 28-fold enrichment for the term “stress response” and related terms, as well as “acetylation,” “ion transport,” “calmodulin-binding,” and “synapse” (Supplementary Spreadsheet 4). Furthermore, IPA showed that differentially expressed IF vs. CR genes converged on Erk1/2 as a central node in the top scoring interaction network involving processes such as “metabolic disease,” “cell death and survival,” and “behavior.” These transcriptional profiles suggest that genes modulated by IF are linked to an enhanced stress response and synaptic plasticity when compared to the CR and AL gene signatures.

**Fig. 3:**
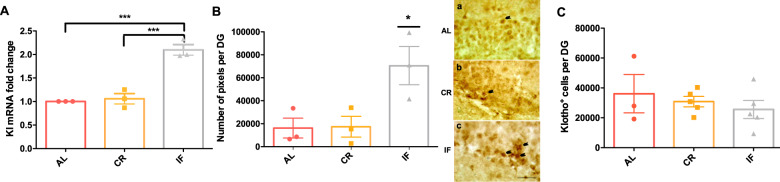
*Klotho* is upregulated by IF. A genome-wide microarray analysis of hippocampal gene expression showed that *Klotho* (Kl) was among the genes found to be upregulated by IF when compared to AL and CR. Validation of the array by RT-PCR showed a two-fold upregulation of Kl in the IF group relative to AL and CR (**A**). Immunostaining for KL revealed a four-fold increase in KL level in the DG of IF mice when compared to CR and AL (**B**). No overall change in the number of KL-expressing cells was found (**C**). Scale bar, 20 μm (**B**). Kl Klotho gene, KL Klotho protein. *p ≤ 0.05; ***p ≤ 0.001.

Among the top genes found to be upregulated by IF when compared to both AL and CR, we here highlight Kl, a gene involved in the suppression of aging phenotypes [27, 46]—a long-known effect of IF [4]. GeneChip analysis of the microarray data showed that Kl was increased by 1.78- and 1.83-fold in the IF group when compared to AL (assigned a value of one) and CR (*F*(2, 6) = 23.27, *p* = 0.0015; CR: 1.06 ± 0.11, *n* = 3; IF: 2.00 ± 0.17, *n* = 3), respectively. Validation of the array by RT-PCR showed a two-fold upregulation of Kl in the IF group relative to both AL and CR (*F*(2, 6) = 45.08, *p* = 0.0002; CR: 1.06 ± 0.11, *n* = 3; IF: 2.10 ± 0.11; *n* = 3) (Fig. 3A). At the gene expression level, the Allen Brain Atlas (http://mouse.brain-map.org/) verified specific expression of Kl in neurogenic niches in the mouse brain (Supplementary Fig. 5). Moreover, immunostaining for KL revealed a four-fold increase in KL level in the DG of IF mice when compared to CR and AL (*F*(2, 6) = 6.68, *p* = 0.003; AL: 16,177 ± 8603, *n* = 3; CR: 17,365 ± 8996, *n* = 3; IF: 70,453 ± 16,617, *n* = 3; expressed as number of pixels per DG) (Fig. 3B). These results indicate that Kl is specifically upregulated by IF in the DG of the adult mouse, despite no overall change in the number of KL-expressing cells (*F*(2,10) = 0.55, *p* = 0.60; AL: 36,082 ± 12,799 cells per DG, *n* = 3; CR: 30,816 ± 3495 cells per DG, *n* = 5; IF: 25,539 ± 6042 cells per DG, *n* = 5) (Fig. 3C).

### Kl is required for hippocampal neurogenesis in vitro

**Fig. 4:**
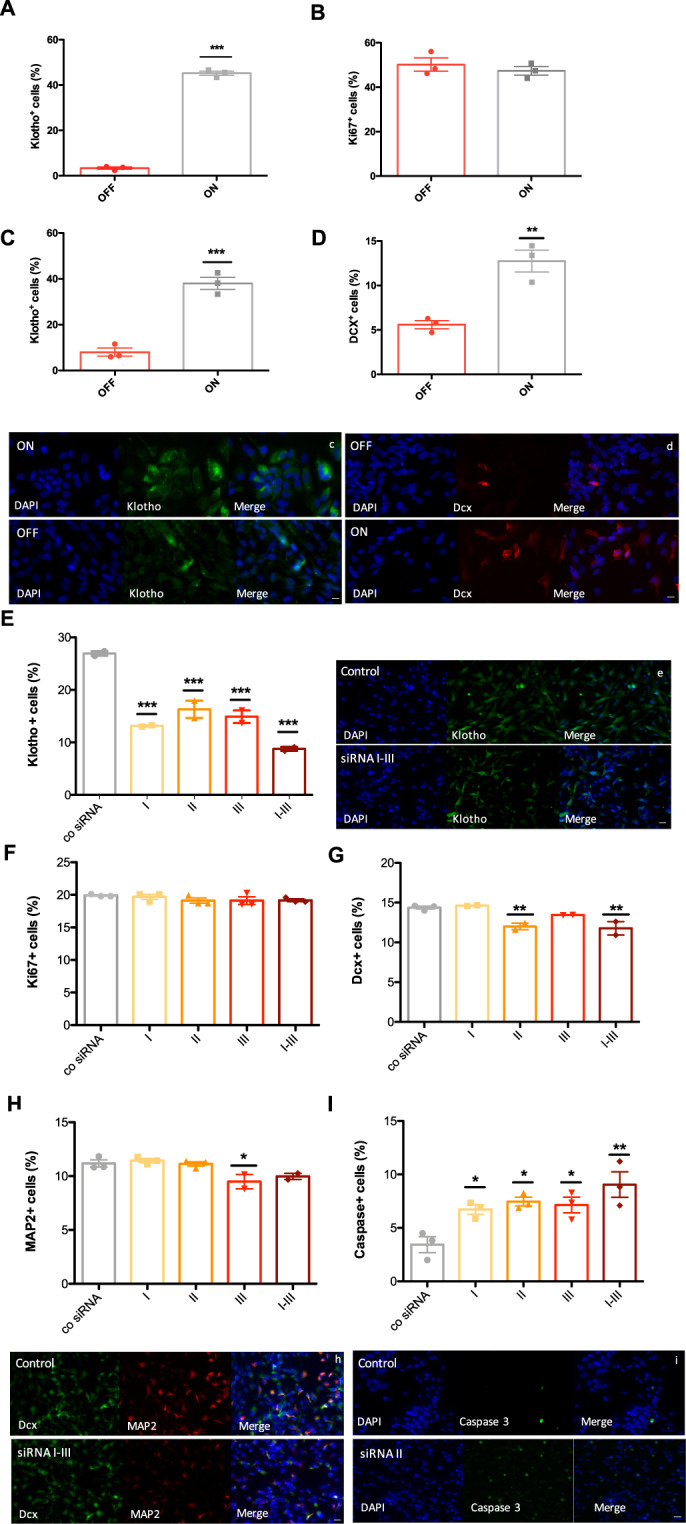
*Klotho* is required for hippocampal neurogenesis in vitro. The human hippocampal progenitor cell line HPC0A07/03A was transduced with a lentivirus to generate a cell line named *Klover* that conditionally overexpressed the secreted form of KL in response to doxycycline withdrawal. After 3 days in proliferation conditions, KL expression in *Klover* ON cells was increased 12-fold when compared to *Klover* OFF cells (**A**), whereas no differences were found between *Klover* ON and OFF conditions regarding levels of cell proliferation as measured by the number of Ki67^+^ cells (**B**). After 7 days of differentiation, the percentage of cells expressing KL was increased 3.8-fold in *Klover* ON cells (**C**; **c**) and the number of DCX^+^ neuroblasts was increased 1.3-fold when compared to *Klover* OFF (**D**; **d**). Three different Kl-binding siRNAs or a mix of all three was used to downregulate Kl in HPC0A07/03A cells. At 7 days post transfection, the proportion of Kl^+^ cells was reduced by 1.65- to 3-fold (**E**). Partial knockdown of Kl did not alter levels of Ki67^+^ (**F**) but the proportion of DCX^+^ cells was significantly decreased with siRNA II and siRNAs I–III (**G**). The percentage of MAP2^+^ cells was significantly decreased following transfection with siRNA I–III only (**H**; **h**), whereas the expression of cleaved-caspase-3^+^ was increased relative to control conditions (**I**; **i**). siRNA studies used an *n* of 2 representing two independent experiments, each with three technical replicates (specifically, the experiments were performed on two independent passages done in three independent plates). Bars in the graph represent the means for each condition. Scale bar, 20 μm. DCX doublecortin, Kl Klotho gene, KL Klotho protein, MAP2^+^ microtubule-associated protein 2, siRNA stealth interference RNA. *p ≤ 0.05; **p ≤ 0.01; ***p ≤ 0.001.

To further investigate the hypothesis that Kl plays a role in regulating hippocampal neurogenesis, we used a controlled system of human hippocampal progenitor cells. Kl was overexpressed and downregulated in the human hippocampal progenitor cell line HPCOA07/03. These cells were transduced with a lentivirus to generate a cell line named *Klover* that conditionally overexpressed the secreted form of KL in response to doxycycline withdrawal (Supplementary Fig. 6). After 3 days in proliferation conditions, KL expression in *Klover* ON cells was increased 12-fold when compared to *Klover* OFF cells, with a total of 45.2% of cells expressing KL overall (*t*(4) = 40.17; OFF: 3.337 ± 0.4856, *n* = 3; ON: 45.19 ± 0.9216, *n* = 3, *p* < 0.0001) (Fig. 4A). No differences were observed in the levels of cell proliferation between *Klover* ON and OFF conditions (*t*(4) = 0.79; OFF: 50.17 ± 2.999, *n* = 3; ON: 47.35 ± 1.949, *n* = 3, *p* = 0.47) (Fig. 4B). After 7 days of differentiation, the percentage of cells expressing KL was increased 3.8-fold in *Klover* ON cells compared to *Klover* OFF conditions, with a total of 40% of cells expressing KL (*t*(4) = 9.35; OFF: 7.934 ± 1.779, *n* = 3; ON: 37.97 ± 2.676, *n* = 3, *p* = 0.0007) (Fig. 4C). In *Klover* ON conditions, the number of DCX^+^ neuroblasts was increased 1.3-fold when compared to *Klover* OFF (*t*(4) = 5.45; OFF: 5.595 ± 0.4613, *n* = 3; ON: 12.74 ± 1.227, *n* = 3, *p* = 0.0055) (Fig. 4D). In *Klover* ON conditions, the proportion of MAP2^+^ neurons and S100β^+^ astrocytes (Supplementary Fig. 7) was non-significantly increased. Together, these results suggest that neuronal differentiation but not cell proliferation is increased when Kl is overexpressed in vitro.

We next downregulated Kl in HPCOA07/03 cells with three different Kl-binding siRNAs (I, II, or III) or a mix of all three (I–III) (Supplementary Fig. 8). At 7 days post transfection, siRNA interference reduced the proportion of Kl^+^ cells by 1.65- to 3-fold (*F*(4, 5) = 49.97, *p* = 0.0003; co-siRNA: 26.91 ± 0.459, *n* = 2; I: 13.14 ± 0.1185, *n* = 2; II: 16.28 ± 1.653, *n* = 2; III: 14.89 ± 1.197, *n* = 2; I–III: 8.766 ± 0.3690, *n* = 2) (Fig. 4E). In line with results from the *Klover* overexpression model, partial knockdown of Kl did not alter the levels of Ki67^+^ (*F*(4, 10) = 1.011, *p* = 0.4465; co-siRNA: 19.91 ± 0.1027, *n* = 3; I: 19.71 ± 0.3805, *n* = 3; II: 19.12 ± 0.3938, *n* = 3; III: 19.12 ± 0.5850, *n* = 3; I–III: 19.16 ± 0.2200, *n* = 3) (Fig. 4F) but the proportion of DCX^+^ neuroblasts was significantly decreased by 16.5% and 18.1% with siRNA II and siRNAs I–III, respectively, when compared to control conditions (*F*(4, 6) = 11.49, *p* = 0.0056; co-siRNA: 14.37 ± 0.1821, *n* = 3; I: 14.62 ± 0.0579, *n* = 2; II:12.00 ± 0.4114, *n* = 2; III: 13.44 ± 0.0277, *n* = 2; I–III: 11.77 ± 0.8411, *n* = 2) (Fig. 4G). Similarly, the percentage of MAP2^+^ cells was significantly decreased by 15.2% following transfection with siRNA I–III only (*F*(4, 8) = 6.261, *p* = 0.0139; co-siRNA:11.17 ± 0.3301, *n* = 3; I: 11.44 ± 0.1923, *n* = 3; II: 11.13 ± 0.2044, *n* = 3; III: 9.481 ± 0.6659, *n* = 2; I–III: 9.966 ± 0.2879, *n* = 2) (Fig. 4H). Furthermore, knockdown of Kl increased expression of apoptotic cell death marker cleaved-caspase-3^+^ by 2- to 2.6-fold relative to control conditions (*F*(4, 10) = 7.331, *p* = 0.0050; co-siRNA: 3.433 ± 0.7457, *n* = 3; I: 6.713 ± 0.4418, *n* = 3; II: 7.452 ± 0.4130, *n* = 3; III: 7.139 ± 0.7304, *n* = 3; I–III: 9.048 ± 1.196, *n* = 3) (Fig. 4I). Together, these results suggest that Kl may play a role in the differentiation and survival of newly born neurons.

### Kl is required in vivo at different stages of AHN in a region-specific manner

**Fig. 5:**
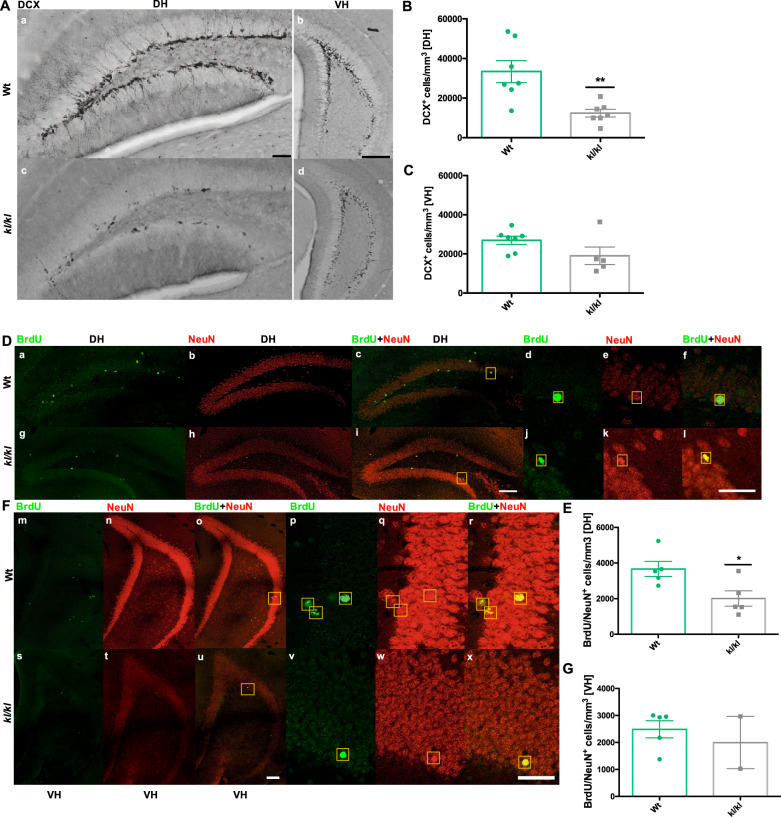
*Klotho* is required for AHN in the dorsal hippocampus. Kl knockout mice (*kl/kl)* displayed decreased number of DCX^+^ cells (**A**) with a significant 2.68-fold reduction being localized to the DH only (**A** [**a**, **c**]; **B**), as opposed to the VH (**A** [**b**, **d**]; **C**). Similarly, co-localization of BrdU and NeuN revealed a reduction in the survival of newborn neurons in the DH (**D**, **F**) and not the VH of *kl/kl* mice (**E**, **F**). Co-localization of BrdU and NeuN revealed a reduction in the survival of newborn neurons in the DH (**D**, **E**) and not the VH of *kl/kl* mice (**F**, **G**). (**D** [**a**–**f**]) = BrdU and NeuN staining in the DH of Wt mice; (**D** [**g**–**l**]) = BrdU and NeuN staining in the DH of *kl/kl* mice. Scale bars, 250 μm (**a**, **c**); 25 μm (**b**, **d**). BrdU bromodeoxyuridine, DCX doublecortin, DH dorsal hippocampus, *kl/kl* Klotho knockout mice, NeuN neuronal nuclei protein, VH ventral hippocampus, Wt wild-type. *p ≤ 0.05; **p ≤ 0.01.

To further investigate the hypothesis that Kl is required for appropriate AHN, the expression of different markers of the neurogenic process was assessed in brain sections from Kl mutant mice (*kl/kl*) [27]. In concordance with the in vitro data, the number of proliferative Ki67^+^ cells was not altered in either the DH or VH of Kl knockout mice (*kl/kl*) (Supplementary Fig. 9). Generation of neuroblasts, however, appears to be strongly dependent on Kl, as shown by the decreased number of DCX^+^ cells in the *kl/kl* group (Fig. 5A) and reflects the in vitro observations following downregulation of Kl. Notably, a significant 2.68-fold reduction in DCX^+^ neuroblasts was localized to the DH only (Wt: 33,410 ± 5547 cells per mm^3^, *n* = 7; *kl/kl*: 12,450 ± 1917 cells per mm^3^, *n* = 7; *p* = 0.003) (Fig. 5B) as opposed to the VH (Wt: 26,920 ± 2090 cells per mm^3^, *n* = 7; *kl/kl:* 19,030 ± 4457 cells per mm^3^, *n* = 5; *p* = 0.107) (Fig. 5C). Similarly, co-localization of BrdU and NeuN revealed a two-fold reduction in the survival of newborn neurons in the DH (Wt: 3661 ± 424.7 cells per DG, *n* = 5; *kl/kl:* 2004 ± 431.2 cells per mm^3^, *n* = 5; *p* = 0.025) (Fig. 5D, E) and not the VH of *kl/kl* mice (Wt: 2485 ± 316.9 cells per mm^3^, *n* = 5; *kl/kl:* 1992 ± 968.9 cells per mm^3^, *n* = 2; *p* = 0.533) (Fig. 5F, G). Differences in dendritic tree arborization of more mature neuroblasts [47] were not found between the two groups, either in terms of branching or length across the dorsal and ventral axis (Supplementary Fig. 10). Previous reports have proposed that the dorsal axis of the hippocampus is linked to cognitive processing, whereas the ventral axis is related to stress, emotion, and affect. Our results demonstrate that Kl is required for AHN in the DH, suggesting a potential mechanism through which IF improves long-term retention memory.

## Discussion

This study addressed whether the beneficial effects of IF on cognition are due to a decrease in total amount of calories consumed or to the increased interval between meals. We demonstrate that, with an overall 10% matched-energy intake, IF in the form of every-other-day feeding is superior to daily CR in enhancing long-term memory performance in mice, and we provide evidence that this enhancement is associated with increased AHN and expression of the longevity gene Kl. Previous studies have already implicated Kl in the regulation of the hippocampal neurogenic process to different extent depending on the age of the mice [32, 33]. Here, we confirm some of these findings in 8-week-old Kl-deficient mice and demonstrate that Kl regulates AHN in a region-specific manner in the dorsal axis of the hippocampus.

IF has previously been demonstrated to enhance learning and consolidation processes [8] and to partly reverse age-associated impairment in motor coordination and memory function in rodents [10]. Similarly, 30–40% CR has been shown to improve learning and memory performance [48], including in a number of different contexts such as aging [7, 49, 50], Alzheimer’s disease (AD) [51], and gastric bypass surgery [52]. To our knowledge, the only previous study comparing the efficacy of IF vs. CR on cognitive performance utilized a transgenic mouse model of AD and revealed that both interventions enhanced locomotion and exploratory behavior and ameliorated age-related memory deficits [20]. IF, but not CR, however, improved cognitive performance without reducing levels of Alzheimer’s pathology, suggesting that IF promotes resilience to pathology and neuronal injury [18, 20]. Notably, many of these reports used 40% CR, whereas in the present study we opted for 10% CR to match the reduced calorie intake in the IF group. The failure of 10% CR to improve memory performance may therefore reflect an insufficient reduction in calorie intake. Ten percent reductions in calorie intake, however, whether achieved by continuous or intermittent reductions, are more likely to be suitable for human compliance [23]. As such, our results indicate the potential of IF over 10% CR to bring about improved cognition. It is worth noting, though, that studies in humans are warranted to determine the most feasible forms of IF that could render improved cognitive performance in this population. This is of particular relevance considering that adherence to the form of IF adopted in the present study might be challenging to promote in human populations. Evaluating the effects of other fasting paradigms, such as adopting longer periods of AL intake between fasting days or time-restricted feeding paradigms, should give us valuable answers as to how to promote realistic adherence to fasting without compromising its positive effects on markers of neuroplasticity [3]. Moreover, in alignment with more traditional regimens utilized in the field, future studies should investigate the effects of 30–40% CR paradigms on cognitive performance and the markers of neuroplasticity investigated here. This will further clarify the potential of daily CR in evoking optimal IF-like effects and the cellular and molecular mechanisms associated with these.

We also demonstrate that both IF and CR increased proliferation and survival of newly born neurons in the hippocampus. Interestingly, an increase in proliferation is not observed in IF paradigms inducing a 30% energy restriction [16, 53], whereas we show for the first time that this effect can occur with a reduction of just 10% calories. Conversely, an extreme 60% CR regimen has been shown to increase neuronal apoptosis and be detrimental to cognition, as measured by levels of Bcl-2 and Bax proteins in the hippocampus and performance in the eight-arm maze, respectively [54]. Therefore, 10% CR may represent an optimal, much less severe value in which increased cell proliferation and survival occurs in the DG, even though not as optimal to AHN as an IF paradigm. While both long-term CR and IF regimens have been associated with improved cognitive functions, lifelong 40% CR has also been linked with increased anxiety in female C57BL/6 mice as measured in the open field and elevated plus-maze tests [50]. A 12-week regimen consisting of either 10% CR or IF could thus be potentially adapted to the clinical setting for the prevention of neuropsychiatric diseases that involve reduced levels of AHN, such as depression [55–58], anxiety [59], and AD [60], overcoming the negative impact on mood of prolonged periods of CR [50].

Our data reveal that only IF animals displayed an increased number of neuroblasts in the DG. These immature cells are known to exhibit different physiological characteristics than those of more mature dentate granule cells, including higher excitability and plasticity [61, 62]. Immature neurons are also thought to contribute to improved retention memory; for example, after 2–8 weeks following acquisition, one report showed a positive correlation between an increased number of neuroblasts in the DG and successful recall in mice [44]. Conversely, long-term memory retention was found to be impaired in mice with reduced populations of immature dentate granule cells [43]. It is possible that the increase in neuroblasts following IF, but not CR, results in a greater number of immature neurons that contribute to improved long-term retention memory. In addition, it is important to remember that a key AHN-dependent function is pattern separation and therefore, for performance in the MWM to be indicative of AHN levels the protocols used must be sensitive enough to test the rodent’s ability to discriminate contexts that are both spatially complex and highly similar [63]. For this, elements such as the inclusion of another acquisition task where the hidden platform has changed places and counting the number of crossings in the expected quadrant as the main read-out of the probe trial phase should be present in the MWM protocol. These elements were not used in our study and this could have hidden the detection of potential positive effects of IF on learning and short-term retention memory. Another question that remains to be answered is on whether the observed enhancing effects of IF on the number of neuroblasts is mediated by increased activity. It is well established that physical exercise increases AHN [34, 64], both through an increase in the number of postmitotic neuroblasts and mature neurons [65]. Besides, evidence from rodent studies using time-restricted feeding protocols demonstrated an increase in locomotor activity [66, 67]. It is therefore plausible to hypothesize that IF could elicit an increase in locomotor activity which, in turn, could both induce an increase in the number of neuroblasts, and their survival into functional mature neurons in the DG. Moreover, gene ontology and pathway analysis of genes differentially expressed in the hippocampi of IF and CR mice revealed marked enrichment for the terms “synapse” and “cognition” as well as involvement of Erk1/2 signaling cascades. These data reveal a transcriptional profile for IF that is indicative of enhanced synaptic plasticity. Interestingly, many of the early studies reporting the benefits of dietary restriction on brain plasticity also showed an upregulation of BDNF and other neurotrophic factors [16]. Importantly, several of these studies employed IF to cause 30–40% reductions in calorie intake; these data and the results herein suggest that increased synaptic plasticity may reflect a key response to IF that also contributes to improved cognition [68, 69]. Interestingly, our microarray analysis also revealed that two genes—namely, Ap2b1 and Camk2a—are similarly regulated in 10% CR and IF conditions (Supplementary Fig. 4), suggesting that these genes could be part of the underlying mechanisms shared by both conditions. This is particularly relevant for Camk2a that has been implicated with neuroplasticity and learning [70].

We also report that the longevity gene Kl was among the top genes significantly upregulated in the IF condition only. Kl has been shown to prevent aging-related symptoms [27, 46] and extend the life span of mice [71], two established effects of IF [4]. Given that IF was also associated with increased levels of AHN, we hypothesized that increased AHN might be occurring via Kl upregulation and undertook assays in a controlled in vitro environment and ex vivo histological analyses of hippocampal slices of Kl mutant mice.

In the in vitro assays, Kl overexpression increased the proportion of neuronal cells but had no effect on the proportion of dividing cells. When Kl was knocked down, neuronal differentiation also decreased, supporting that Kl plays a role in neuronal fate determination. The proportion of apoptotic cells was also significantly increased under Kl knockdown and aligned with previous evidence supporting Kl’s ability to modulate cell death [72]. Furthermore, the Kl-binding siRNAs used here prevented the transcription of secreted KL RNA; it is known that secreted KL increases resistance to oxidative stress [73] and inhibits tumor necrosis factor α and tumor growth factor β signaling pathways [74]. In addition, secreted KL promotes longevity by inhibiting insulin/IGF-1 signaling [55]. Kl has also been shown to participate in glutamatergic signaling in the brain [25, 75], and glutamate is known to influence neurogenic stages such as neuronal commitment [76]. In the present study, “stress response” was one of the strongest enrichment terms for IF-linked genes when compared to CR, suggesting an upregulation of adaptive cellular stress response pathways that promote neuronal survival [12]. Taken together, our findings suggest the convergence of multiple mechanisms that result in increased AHN, although future in vitro studies investigating the effects of Kl on hippocampal neurogenesis using siRNA transfection should ideally use an increased number of biological replicates.

In alignment with our in vitro assays, the histological data of *kl/kl* mice showed that cell proliferation did not require Kl but early cell fate into neuroblasts and AHN were significantly dependent on appropriate levels of KL. Notably, this finding was only observed in the DH, the axis most implicated in cognitive processing and spatial memory [77]. Although previous studies have demonstrated that Kl is important for AHN [32, 33], to our knowledge this is the first time that a gene has been proposed to regulate AHN specifically in one axis of the hippocampus. This finding is aligned with previous reports that showed Kl knockout mice exhibit impaired long-term memory recall without changes in emotionality [25, 78]. Conversely, overexpression of Kl in mice resulted in significantly better memory performance via augmentation of synaptic GluN2B unit levels in the hippocampus and cortex [25]. Improved learning and memory have also been found in mice 6 months following a single injection of the secreted isoform of KL [79]. In healthy humans, a variation in the Kl gene has also been associated with greater cortical volume and better cognition [80], as well as reduced risk for AD [81].

IF elicits adaptive cellular responses that are integrated in the periphery and within organs in a manner that improves glucose regulation, increases stress resistance, and suppresses inflammation [82]. Therefore, our data showing that IF affects AHN with the expected behavioral consequences are likely just one of the mechanisms that contribute to IF’s role in cognition. Equally, Kl is expressed in multiple cell types, circulating in the blood, the cerebrospinal fluid, and provides the potential that Kl has widespread actions that likely affect cognition by multiple mechanisms than just through increasing AHN. Finally, Kl is unlikely the sole mediator by which IF regulates AHN. For example, IF has been recently shown to alleviate diabetes-induced cognitive impairment via the gut microbiota [83]. It is still to be definitively determined whether Kl-mediated increases in AHN following IF improve cognitive performance. One way to fully answer this hypothesis would be to apply an IF regimen to Kl mutant mice followed by an investigation of cognitive function and AHN. This experiment, however, would probably need to be done with conditionally knocked out animals, as the *kl/kl* mice utilized here are unlikely to survive full days of food restriction due to severe hypoglycemia that is a known cause of death in mice [84, 85]. Besides, Kl mice die prematurely at approximately 8–9 weeks of age [27] that was the starting age of our diet experiments. One possibility for future studies could be a careful titration of IF and CR in Kl mice and the consideration of an earlier start of the dietary regimen.

Another topic that deserves special attention is that of sex differences in neurogenesis. It is known that AHN is differentially regulated in males and females [86]. While we have used both sexes in the histological analyses of Kl mice, we only used female mice in the diet experiments. Future studies should include females and males so that a more accurate picture of the behavioral, cellular, and molecular effects of energy restriction for both sexes is depicted. Nevertheless, the data presented here are relevant especially given that the activity of new neurons as recruited during spatial memory retrieval is similar across sexes [87].

In conclusion, we showed that IF is more effective in improving long-term memory retention and generating more newborn neurons in the DG when compared to 10% CR. Moreover, we found that Kl, the longevity gene, is upregulated by IF only and that Kl is required for appropriate hippocampal neurogenesis in vitro and in vivo, especially in the DH. Our findings suggest that IF has the potential to be a potent cognitive enhancer, a finding that holds promise for use in humans. The search for the molecular pathways regulated by Kl in the hippocampus might also shed light on important pharmacological targets whose activation may mimic the beneficial effects of fasting on mental health.

## Supplementary information

Supplemental material

Data Set 1

Data Set 3

Data Set 2

Data Set 4
