# Supplementary material for: Intermittent fasting enhances long-term memory consolidation, adult hippocampal neurogenesis, and expression of longevity gene Klotho
Source: Mol Psychiatry. 2021 May 25;26(11):6365–79. doi: 10.1038/s41380-021-01102-4 (PMC8760057; doi:10.1038/s41380-021-01102-4)
Supplement: Supplementary file 1 — Supplemental material [file 41380_2021_1102_MOESM1_ESM.docx]

**Supplementary Information**

**Intermittent fasting enhances long-term memory consolidation, adult hippocampal neurogenesis and expression of longevity gene Klotho**

Gisele Pereira Dias^^^, Tytus Murphy^^^, Doris Stangl^^^, Selda Ahmet, Benjamin Morisse, Alina Nix, Lindsey J. Aimone, James B. Aimone, Makoto Kuro-O, Fred H. Gage, Sandrine Thuret.

**Animals used in the diet experiments.**A total of 75 female C57BL6 mice were randomly allocated to one of three groups: *ad libitum* (AL), 10% daily calorie restriction (CR) and every-other-day feeding (Intermittent Fasting, IF). Following three months of either AL, 10% CR or IF regimen, all animals were fed *ad libitum* for 5 days as a wash-off period prior to tissue collection. The figure below details the distribution of animals for behavioral testing, gene expression, stereology microscopy and stereology/ confocal microscopy:

 
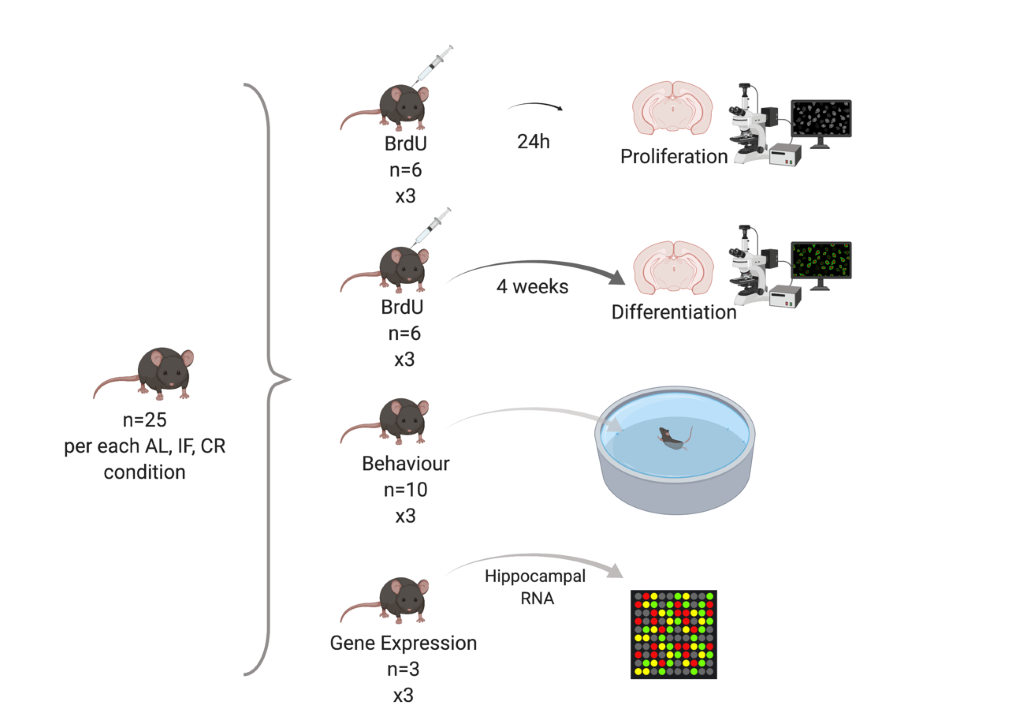


**Supplementary Figure 1: Mice population for the diet experiments.**Each group (AL, CR and IF) was composed of 20-25 female C57BL6 mice, of which 5 AL, 10 CR and 10 IF were tested in the Morris water maze (MWM), three had their hippocampi dissected and RNA extracted for gene expression analysis, and 12 were used for histological analysis (six were sacrificed 24 hours after last BrdU injection; 6 were sacrificed four weeks after the last BrdU injection). Sample size was estimated from previous studies (1, 2), no randomization was used. AL = *ad libitum*; BrdU = bromodeoxyuridine; CR = calorie restriction; IF = intermittent fasting.

**Generation of Kl mice.** The generation of Kl mice followed procedures described previously (1). Briefly, Kl mice (*kl/kl*) result from the crossing of Kl^+/-^ mice generated by insertion of a mutation disrupting the 5’ promoter region of the Kl gene. The resulting wild-type littermates were used as control animals.

**Immunostaining of free-floating sections (BrdU) and immunofluorescence for BrdU+NeuN.**For each diet group and Kl brain, one in six series of sections were transferred into one well of a six-well plate. Staining procedures followed those previously described (2). The procedures for staining Kl mice brains against BrdU and BrdU+NeuN occurred as follows:

**BrdU.**One-in-six series of sections from BrdU-injected mice (n= 2-5 brains/group) were washed, exposed to 1% hydrogen peroxide for 30 minutes, washed again and kept for 30 minutes at 37 °C in 2 N HCl, following 2 x 10-minute rinses in 0.1 M borate buffer at room temperature (RT). Sections were washed and exposed to 3% normal goat serum (NGS) (Vector Laboratories, S-1000) for 1 hour. Sections were incubated overnight with anti-BrdU primary antibody (Serotec OBT0030; 1:1,000 in 3 % NGS), washed, and incubated with goat anti-rat secondary antibody (Vector Laboratories, BA-9400; 1:250 in 3 % NGS) for 2 hours. Sections were washed and incubated in ABC for 2 hours. Finally, sections were washed and incubated in ABC complex (Vector Laboratories, PK-6100, Vectastain® Elite ABC-Peroxidase Kits; 1:1,000) for 2 hours. Following washes, the reaction was revealed by DAB (Sigma, D5637-5G). Slides were mounted in DPX (Sigma, 44581).

**BrdU+NeuN.**Sections (n= 2-5 brains/group) were washed and kept for 30 minutes at 37 °C in 2 N HCl, following 2 x 10-minute rinses in 0.1 M borate buffer at RT. Sections were washed and exposed to 3% normal goat serum (NGS) (Vector Laboratories, S-1000) for 1 hour. Sections were incubated overnight with anti-BrdU and anti-NeuN primary antibodies (respectively, Serotec OBT0030, 1:250; Chemicon IHCR1001-6, 1:10 in 3% NGS), washed 2x 15 min with TBS + 1x 15 min with TBS-T, and incubated with Alexa Fluor 488 goat anti-rat and Alexa Fluor 594 goat anti-Mouse (respectively, Invitrogen A11006, 1:250; Invitrogen A-11005, 1:250) for 2 hours protected from light. Sections were washed and mounted with ProLong Gold Antifade Reagent with DAPI (Invitrogen P36935). Washes represent 3 x 5-minute rinses in TBS except when stated otherwise.

**Immunostaining of free-floating sections**

**Doublecortin (DCX)/ Klotho (KL).**Sections (DCX, n=4 AL; n=7 CR; n=9 IF/ KL, n=3/group) were incubated in 1% H202 in TBS for 40 minutes at RT. Next, sections were rinsed 3x for 5 minutes in TBS before being blocked in 15% normal goat serum (NGS) for KL staining, or NRS for DCX staining, in TBS-T (TBS + 0.3% Triton-X) for 30 minutes. Incubation with primary antibodies (x-Klotho (E-21), Santa Cruz, SC-22220; 1:500 or x-DCX, Abcam, Ab18723; 1:10,000) was carried out in a dilution of 10% NS in TBS-T at 4°C overnight. The following day sections were rinsed 3x for 5 minutes in TBS and incubated with the appropriate biotinylated secondary antibody diluted in 10% NS in TBS-T for 2 hours. Sections were then rinsed again 3x for 5 minutes and incubated with in Vectastain ABC/*Elite* Standard Kit (Vector Laboratories), diluted at 1:1.000 in TBS. Following washes, the reaction was revealed by DAB (Sigma, D5637-5G). Slides were mounted in DPX (Sigma, 44581).

**Ki-67/ DCX.**One-in-six series of sections from non-BrdU injected Kl mice and Wt brains (Ki-67: n=5-7 brains/group; DCX: n=7 brains/group) were separately processed for each of these markers. Sections were washed, exposed to 1% hydrogen peroxide for 30 minutes, washed and kept for 2 hours in 15% NGS. Sections were incubated overnight with anti-Ki-67 (ABCAM, AB15580; 1:1,000 in 10% NGS) or anti-DCX primary antibody (Abcam, AB18723; 1:1,000 in 10 % NGS). Sections were washed and then incubated with goat anti-rabbit biotinylated secondary antibody (Vector Laboratories, BA-1000; 1:1,000 in 10 % NGS). Finally, sections were washed and incubated in ABC complex (Vector Laboratories, PK-6100, Vectastain® Elite ABC-Peroxidase Kits; 1:1,000) for 2 hours. Following washes, the reaction was revealed by DAB (Sigma, D5637-5G). Slides were mounted in DPX (Sigma, 44581).

The table below contains information on the codes and dilutions of the primary and secondary antibodies used in the histological analyses of the present study.

| **Primary antibodies** | **Dilution** | **Secondary antibodies** | **Dilution** |
| --- | --- | --- | --- |
| **x-BrdU: SerotecOBT0030** | 1:1,000 | Anti-rat, BA-9400  (Vector Laboratories) | 1:250 |
| **x-BrdU: SerotecOBT0030; x-NeuN:Chemicon IHCR1001-6** | 1:250; 1:10 | Anti-rat 488, Anti-mouse 594  (Invitrogen A11006;  Invitrogen A-11005) | 1:250; 1:250 |
| **x-DCX: Abcam, AB18723** | 1:10,000; 1,000 | Anti-rabbit BA-1000 (Vector Laboratories) | 1:1,000 |
| **anti-Ki-67: Abcam, AB15580** | 1:1,000 | Anti-rabbit BA-1000 (Vector Laboratories) | 1:1,000 |

**Supplementary Table 1: Antibodies used for immunohistochemistry of diet and klotho knockout mice.**BrdU=bromodeoxyuridine; DCX= doublecortin; NeuN= neuronal nuclei.

**Stereological and confocal analysis.**The optical fractionator (3) method of stereology was used to count the number of DAB-revealed positive cells in sections of AL, CR and IF brains (BrdU; DCX), as well as in *kl/kl* and wild-type (Wt)brains (DCX).

In the case of *kl/kl* and Wt brains, the volume of the DG of BrdU- and Ki-67-stained sections was calculated by the Cavalieri Estimator method using a semiautomatic stereology system (StereoInvestigator, MicroBrightfield) and a 2.5x objective. BrdU^+^ and Ki-67^+^ cells were manually counted in a Zeiss Axioimager Z1 microscope using a 40x objective, and the total estimation was made by summing up the number of positive cells and adjusting to the estimated volume multiplied by the section thickness (40 µm) and series of sections collected (3), so that a value of cells/mm^3^ could be achieved.

***IF is more effective than CR in promoting long-term memory retention and increasing the number of neuroblasts in the dentate gyrus***

**Septo-temporal analysis of DCX^+^ cells.** Detailed methods for immunostaining of free-floating sections against DCX can be found in the Materials & Methods section of this article. Similarly to the other septo-temporal analyses undertaken in this study, the criteria used to differentiate the DH and the VH followed those described previously (6). Brain slices within -1.06 to-2.06 mm relative to bregma were used for the DH, and those containing coordinates -3.08 to-3.80 mm were used for the VH, in accordance with the Paxinos and Franklin’s brain atlas (4).

Interestingly, the IF group had a significantly increased number of neuroblasts both in the dorsal (F(2, 17) = 6.131; p = 0.0099; AL, n=4; CR, n=7; IF, n=9) and ventral hippocampus (F(2, 17) = 5.785; p = 0.0121; AL, n=4; CR, n=7; IF, n=9). Post hoc analyses revealed that differences were observed only between AL and IF; no differences were found between the IF and 10% CR groups. This suggests that other mechanisms that not those directly regulated by Kl may also play a critical role in modulating AHN under IF, especially in the ventral hippocampus:


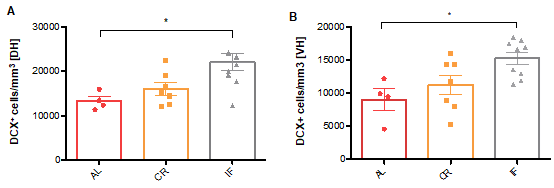


**Supplementary Figure 2A**: **Septo-temporal distribution of DCX^+^ cells in the DG of diet mice.** There was an increased number of DCX^+^ cells both in the DH (A) and VH (B) of IF animals, as compared with AL and CR. AL = *ad libitum*; CR = calorie restriction; IF = intermittent fasting; DH = dorsal hippocampus; VH = ventral hippocampus.

**Dendrite quantitative morphometric analysis.**Granule neurons (Diet brains: 32 neurons, n=5 AL/ 64 neurons, n=8 CR; 64 neurons, n=8 IF / *kl/kl* x Wt brains: DH: 54 Wt/ 29 Kl; VH: 44 Wt/ 34 *kl/kl*; n=7 brains/group) expressing DCX and exhibiting a more mature dendritic tree (classified as types C-D or E-F according to previously proposed categorization (5) were imaged using a 40x objective, and micrographs were acquired using a Zeiss AxioCam MR Rev3 camera, following procedures previously described (6). Dendrites were traced, categorized and analyzed using the NeuronJ plugin for ImageJ (http://rsbweb.nih.gov/ij/) by a blinded experimenter. The criteria used to differentiate the DH and the VH followed those described previously (7). Specifically, brain slices within -1.06 to-2.06 mm relative to bregma were used for the DH, and those containing coordinates -3.08 to-3.80 mm were used for the VH, in accordance with the Paxinos and Franklin’s brain atlas (4).

As shown in Supplementary Figure 2B, no differences were found between neuroblasts in AL, CR and IF mice with regard to either total number of dendritic branches per neuroblast (F(2, 187)= 2.613, p= 0.0760; branches/neuroblast: AL,7.710±0.5271, n=31; CR, 6.810±0.2999, n=79; IF, 7.900±0.3960, n=80) or dendritic length (F(2, 157)= 2.351, p= 0.0986;length(μm)/neuroblast: AL, 332.5 ±25.19μm, n=32; CR, 303.7±13.62μm, n=64; IF, 352.6±17.20μm, n=64):


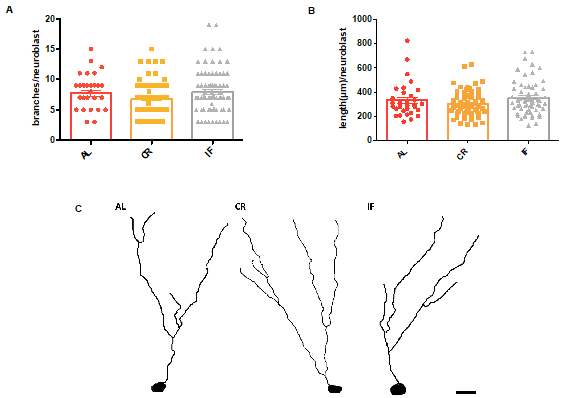


**Supplementary Figure 2B: Total number of dendritic branches per cell and dendritic length in neuroblasts of AL, CR and IF mice.**No differences were observed between groups with regard to either total number of dendritic branches per cell (A) or dendritic length (B). Scale bar = 25 um (C). AL = *ad libitum*; CR = calorie restriction; IF = intermittent fasting.

***Genome-wide expression analysis reveals*Klotho*is upregulated by IF***

**GeneChip analysis.**A list of genes that could be involved in the modulation of AHN was obtained using Affymetrix GeneChip technology. Briefly, genes were chosen on the basis of 1) most pronounced fold change in expression in the CR-and IF-fed groups relative to the AL control population, 2) the highest number of methods in which they appeared, and 3)background research carried out on potential genes, which included using PubMed for verifying their function in the central nervous system, as well as examining their *in situ* hybridization expression patterns using the Allen Brain Atlas (www.brainatlas.org) to confirm whether they were expressed in the dentate gyrus  (DG).

For each of the three dietary groups, the total RNA from three different mice was independently extracted from the whole hippocampi as done previously (2). The mRNA of each mouse was amplified, labelled and hybridized on Affymetrix GeneChip Mouse Genome 430 2.0 Arrays. The nine chips (three per each dietary group) were analyzed using four different complementary analytical tools and the levels of gene expression were compared between all three dietary groups. The remaining RNA was stored and used for quantitative RT-PCR to validate genes of interest.

Briefly, the data were pre-processed using the Affymetrix Microarray Analysis Suite (MAS) and subsequently four analytical methods were used to analyze the GeneChip data: RMA (8, 9) and dChip (10), which both give an estimation of expression level and fold change, and the ‘PM-MM’ and ‘PM-only’ versions of the Drop Method (11), which return a confidence that there was a difference in expression, independent of magnitude. Relatively loose criteria were initially used to filter the data (to minimize analytical false negatives), but genes were required to pass these criteria for each of the four methods to be considered further (four out of four methods) to minimize analytical false positives.

A fold change of 1.2 along with a t-test p-value of less than 0.05 was used in RMA and dChip, and a confidence of 50% or higher was used in the Drop Method. Most of the genes returned as candidates had fold changes and confidence values well above these thresholds. The use of loose criteria over multiple methods also allowed genes that met criteria in some but not all of the methods (i.e., three of four methods) to be reviewed with the understanding that they were more likely to be false positives.  The combination of these methods has been shown to reduce both false positives and false negatives (11, 12).

**Pathway analysis.**Differentially expressed genes between IF and CR, as identified by three of the four analytical methods above, were taken forward for pathway analysis using DAVID (https://david.ncifcrf.gov/) and ingenuity pathway analysis (IPA, http://www.ingenuity.com, Qiagen, USA). In both cases a mouse genome was used as the background reference. Networks and pathways with a P-value < 0.05 were regarded as significant.


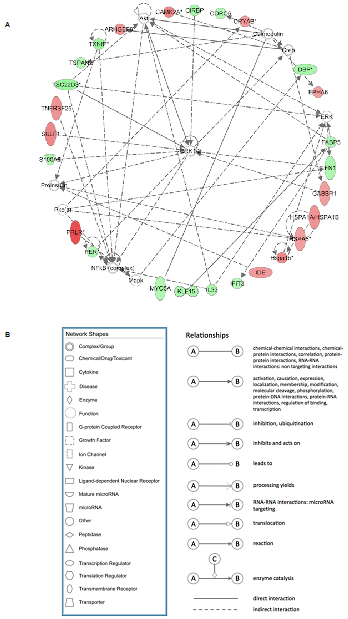


**Supplementary Figure 3: Top network formed by differentially expressed genes in response to intermittent fasting (IF) and calorie restriction (CR):** 176 differentially expressed genes were mapped from 186 probes in response to IF and CR and then analyzed using Ingenuity Pathway Analysis (IPA) to assess network relationships between candidate genes. **A** Top network shown here and associated functions included ‘metabolic disease’, ‘cell death and survival’ and ‘behavior’. Magnitude of fold change in gene expression reflected on continuous color scale for upregulated (red to pink) to downregulated genes (dark to light green). Inferred molecular interactions identified by IPA are shown in gray. **B** Legend description for figure **A,** describing the types of molecules involved and their relationships with one another.

**Primer design.**Once the genes of interest had been chosen (Supplementary Figure 4), their cDNA sequence was obtained in FASTA format using the site http://www.ncbi.nlm.nih.gov/. Each sequence was then used to design primers (http://pathogene.swmed.edu/rt_primer/; see Supplementary Table 2 for chosen genes and their primer sequences). Parameters were set to give a primer size between 18 and 27 base pairs (bps) and an optimum GC nucleotide content of 50% to prevent self-hybridization in the G/C region. The parameters were also adjusted to give an amplicon size of 100-250 bps and a primer melting temperature of 60°C. To ensure that the primers would be specific for that gene of interest, both the forward and the reverse primer sequences were blasted against the mouse genome using the NCBI Nucleotide Blast website: http://www.ncbi.nlm.nih.gov/BLAST.

Forward and reverse primers designed for each gene were ordered from Sigma. Primers were dissolved in a specified amount of water to give a concentration of 100 µM. From this, an equal amount of forward and reverse primers wascombined and diluted with DEPC treated water (SIGMA, 95284) to give a final concentration of 2 µM. Primers were required to be at this concentration in order to be used in RT Q-PCR experiments.

| **Primer** | **Forward** | **Reverse** |
| --- | --- | --- |
| **Ap2b1** | GTGGTCTGAATGACCTGTTTGA | TTAGCCTTTACTGCAGGTAGCC |
| **Agxt2l1** | CTCCAAGAGTGGAACTGTGTTCT | GGTCTGAGCATACACCATTTCTC |
| **Aqp1** | CACTGTGCCCTTAACCACATT | CTGTGATATGCCAGTGGTCAGT |
| **Camk2a** | ACACGTGGAAGGAGGAAGTCT | CACAGCGTGAGAAAGAGCAG |
| **Cirbp** | GCATCAGATGAAGGCAAGC | CTTCGGAGATCTGCCCATAC |
| **Enpp2** | TGTATGACCCTGTCTTTGATGC | TTGGTGGCTGTAATCCATAGC |
| **Kl** | CCAAAGTCTGGCATCTCTACAAC | AGCCTAGCACAAAGTCAAGAGAC |
| **Prlr** | CTTCCTGCTCTGTCTCACTCACT | CGTTCTTTAGTTCTGCTGGAGAG |
| **Sgk** | ATCTGCACTCCCTAAACATCGT | TTGCAGAGCCCAAAGTCAG |
| **Tle1** | TGCATGTGAACAAGCCTGA | CCAGTACTCACAAACCATTTGC |

**Supplementary Table 2: Chosen genes and their primer sequences.** Ap2b1 (Adaptor-related protein complex 2, beta 1 subunit); Agxt2l1 (Alanine-glyoxylate aminotransferase 2-like 1); Aqp1 (Aquaporin 1); Camk2a (Calcium/calmodulin-dependent protein kinase II alpha); Cirbp (Cold inducible RNA binding protein); Enpp2 (Ectonucleotide pyrophosphatase/phosphodiesterase 2), Kl (Klotho); Prlr (Prolactin receptor); Sgk (Serum/glucocorticoid regulated kinase); Tle1 (Transducin-like enhancer of split 1, homolog of Drosophila E(spl)). http://www.ncbi.nlm.nih.gov/BLAST (12).

**RT-QPCR.**RT-QPCR was carried out on each of the chosen genes to measure their level of expression in the different dietary groups. First, cDNA was made using oligodT using the Omniscript Qiagen Kit from the total RNA previously extracted from hippocampi of each dietary group (protocol for reverse transcription can be found in the “Omniscript Reverse Transcription Handbook,” which can be downloaded from www.qiagen.com*).* The cDNA for AL, CR and IF groups were then purified using the PCR purification Qiaquick Kit (protocol can be obtained from the “Qiaquick Spin Handbook” from www.qiagen.com). The target concentration after purification of cDNA was 20 ng/5 μl, which is the desired template concentration for RT Q-PCR.

RT Q-PCR was carried out using white 96-well PCR plates (BioRad). Each well contained 20 μl of the reaction mixture that contained 2 μl of primer mix (consisting of 2 μM of both forward and reverse primers of the gene of interest), 3 μl double distilled water, 5 μl cDNA template and 10 μl 2x Real Time PCR Master mix (containing Taq polymerase, buffer and SybrGreen; primerdesign.co.uk). Pilot studies were carried out to ensure that the primers worked before using them to measure the level of gene expression in the cDNA of each dietary group (AL1-AL3, CR1-CR3 and IF1-IF3, i.e., each biological triplicate). When measuring the level of gene expression, primers for one gene of interest were run against all nine cDNA templates (AL1-AL3, CR1-CR3 and IF1-IF3) so that any biological errors could be accounted for. Additionally, on the same plate one housekeeping gene, phosphoglycerate kinase-1 (Pgk-1), was tested on each cDNA template. On each PCR plate both the gene of interest and the housekeeping gene had duplicates and a control sample (template replaced with double distilled water) so that experimental errors could be identified.

The efficiency and cycle time (Ct) were obtained for each gene (including the house-keeping gene Pgk-1) and its experimental duplicate for all dietary groups. For each group, the average efficiency and average Ct values were determined from each gene and its duplicate, thus reducing the size of any experimental errors such as differences in cDNA concentration and pipetting. These values were then used to calculate the total average efficiency and Ct in each group, i.e., average efficiency and Ct values obtained from three independent biological samples in the AL group (AL1-3), the CR group (CR1-CR3) and the IF group (IF1 –IF3). These values were then used to calculate the fold change in expression of each gene in the CR and IF fed groups using the Pfaffl equation. The AL group was assigned a value of one, and any fold change in expression of a gene was calculated relative to this:


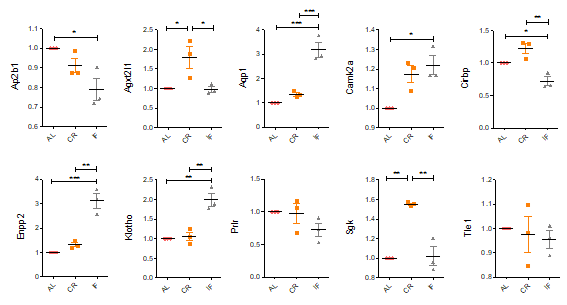


**Supplementary Figure 4: Expression of chosen genes (expressed as fold-change relative to *ad libitum* (AL)).**Ap2b1 (Adaptor-related protein complex 2, beta 1 subunit); Agxt2l1 (Alanine-glyoxylate aminotransferase 2-like 1); Aqp1 (Aquaporin 1); Camk2a (Calcium/calmodulin-dependent protein kinase II alpha); Cirbp (Cold inducible RNA binding protein); Enpp2 (Ectonucleotide pyrophosphatase/phosphodiesterase 2), Kl (Klotho); Prlr (Prolactin receptor); Sgk(Serum/glucocorticoid regulated kinase); Tle1 (Transducin-like enhancer of split 1, homolog of Drosophila E(spl)).http://www.ncbi.nlm.nih.gov/BLAST (13). Note that genes Ap2b1 and Camk2a are similarly regulated by 10% CR and IF and could be part of the shared mechanisms between both conditions. Others are specifically regulated by 10% CR(Agxt211 and Sgk) and IF (Aqp1, Enpp2 and Kl) and likely participate in the differential effects of each regimen. AL= *ad libitum*; CR= calorie restriction; IF= intermittent fasting. *P ≤ 0.05; **P ≤ 0.01; *** P ≤ 0.001.

Further evidence supporting a potential role for Kl in hippocampal function came from the Allen Brain Atlas (http://mouse.brain-map.org/),where specific expression of Kl in neurogenic niches in the mouse brain was verified, as shown by in situ hybridization (ISH):


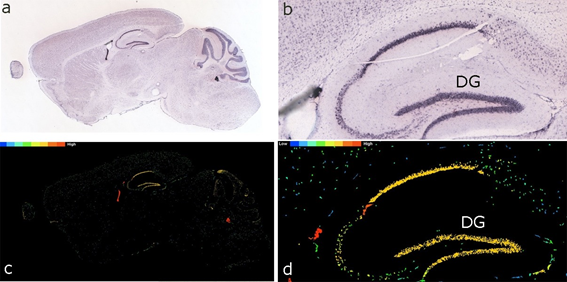


**Supplementary Figure 5: Expression of the Klotho gene in the mouse brain, as verified by ISH in the Allen Brain Atlas (http://mouse.brain-map.org/).**Note the high expression of this gene in neurogenic niches such as the DG. DG = dentate gyrus; ISH = in situ hybridization.

**Klotho*is required for hippocampal neurogenesis*in vitro**

**Generation of Kl over-expressing cell line (***Klover***)**

**Cloning of secreted Kl into the pVLX-Tight-Puro vector.**The human Kl secreted plasmid in pcDNA3.1/V5/His-TOPO back bone (17713) was purchased from Addgene (Cambridge, USA) and was then cloned into the pLVX-Tight-Puro vector (ClonTech 632162) using the enzymes BamHI and Xba1 (NEB), as follows. The vector and the target gene plasmid were digested using the same enzymes to create matching sticky ends. The cutting site of the enzyme Xba1 (T/CTAGA) is blocked by overlapping dam methylation. Dam methylase–methylation occurs at the N6 position of the adenine in the sequence GATCT (14, 15). To be able to digest the blocked Xba1 site, the plasmids were transferred into StellarTMdam-/dcm- competent cells (Clontech) following manufacturer’s instructions to prevent blocking of the site through dam methylation. The plasmids were extracted using the Sigma GenElute miniprep (PCN70-1KT) following manufacturer’s instructions. To isolate the specific fractions, vector and insert, the plasmids were then subjected to a double digest with BamHI and Xba1 using 5 ug of DNA. The products of the digest were concentrated using QIAprep Spin Miniprep Kit (Qiagen) following manufacturer’s instructions, checked for quality on an ethidium bromide gel and separated using a Crystal blue gel. The insert and vector were then purified from the gel using QGbuffer (Qiagen) and the GeneJet Plasmid Miniprep kit (Fermentas) following manufacturer’s instructions. For ligation, the insert DNA and the vector DNA were mixed with 1 μl ligase (NEB) and 2 μl 10x ligation buffer (NEB) in a total reaction volume of 10 μl and incubated for 30 minutes at RT. The plasmid was then transformed into dam-/dcm- competent cells and sequenced for the correct insertion of the target gene. The plasmid was then extracted and purified using Pure Yield Plasmid Maxiprep (Promega) following manufacturer’s instructions. A glycerol stock of the E. Coli carrying the Kl secreted pLVX-Tight-Puro Plasmid was stored at -80°C.

**Sequencing of the Kl secreted pVLX-Tight-Puro plasmid.**The plasmid was amplified using the BigDye® Terminator v1.1 Cycle Sequencing Kit (Applied Biosystems) using the primers and the conditions in the table below on the BioRad Opticon 2.

| **Primers used for sequencing the correct insertion of the Kl secreted insert into the pVLX-Tight-Puro vector:** |
| --- |
| ***Forward primer*** |
| **CMV: CGCAAATGGGCGGTAGGCGTG** |
| **Fw1: AGGCCCTTTCGTCTTCACTC** |
| **Fw2: TAGCCAGCGACAGCTACAAC** |
| **Fw3: CATCGACAACCCCTACGTG** |
| ***Reverse primer*** |
| **BGH: TAGAAGGCACAGTCGAGG** |
| **Rev1: GGGGAACTTCCTGACTAGGG** |
| **Rev2: TAGGGCTTGGTGAGACTGCT** |
| **Rev3: GGTCCAAAGCAAAGAGCAAA** |
| **PCR protocol used for amplifying the Kl secreted pLVX-Tight-Puro plasmid:** |
| **96.0°C 01:00 min**  **96.0°C 00:08 min**  **50.0°C 00:07 min**  **60.0°C 01:30 min**  **Go to line 2 for 14 more times**  **96.0°C 00:08 min**  **50.0°C 00:07 min**  **60.0°C 01:45 min**  **Go to line 6 for 4 more times**  **96.0°C 00:08 min**  **50.0°C 00:07 min**  **60.0°C 02:00 min**  **Go to line 10 for 4 more times**  **10.0°C 16:00:00**  **End** |

**Supplementary Table 3: Sequencing of the Kl secreted pVLX-Tight-Puro plasmid.**Primers and the conditions used on the BioRad Opticon.

To remove unincorporated nucleotides, the sequencing PCR product was cleaned up using the Ethanol/EDTA precipitation protocol following manufacturer’s instructions (Applied Biosystems), resuspended in Hi-Di Formamide (applied Biosystems) and assayed on an Applied Biosystems 3130 genetic analyzer. The resulting sequence electropherograms were then analyzed using BioEdit and Codon Code Aligner software**.**

***Klover* assay.**To investigate the hypothesis that Kl is required for hippocampal neurogenesis in a controlled system, Kl was over-expressed in the human hippocampal progenitor cell line HPCOA07/03 (Johansson et al., 2008). These cells were transduced with a lentivirus to generate a cell line named *Klover* that conditionally over-expressed the secreted form of KL upon exposure to doxycycline. Cells were then kept under either proliferative (three days) or differentiating (seven days) conditions.


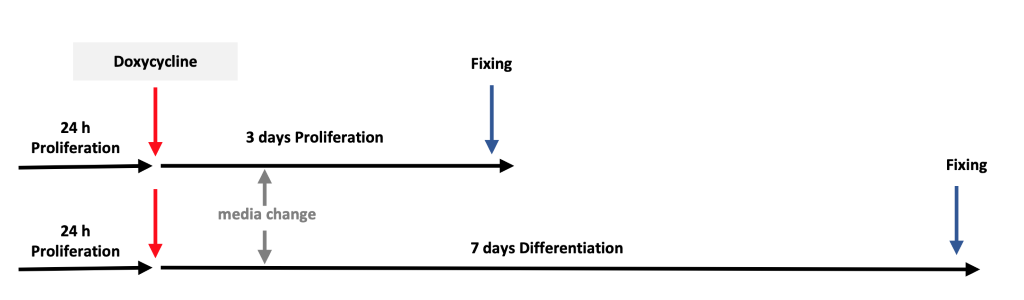


**Supplementary Figure 6: Timeline for the assay using *Klover* HPCOA07/03 cells.** Cells were treated with 1 μg/ml Doxycycline 24 hours after seeding. Media was changed after 24 hours to doxycycline-free proliferation condition for three days or under differentiation condition for seven days.

**Expression of mature neurons and astrocytes in *Klover* ON conditions.** Detailed methods for immunocytochemistry can be found in the Materials & Methods section of this article. As shown in the figure below, the proportion of mature neurons and astrocytes was non-significantly increased in *Klover* ON conditions after seven days of differentiation, as revealed by immunostaining for MAP2 (OFF: 8.451±0.9536, n=3; ON: 28.71±9.815, n=3; p=0.1091; Supplementary Figure 7A) and S100β (OFF: 13.57±0.6927, n=3; ON: 29.75±7.063, n=3; p= 0.0848; Supplementary Figure 7B), respectively:


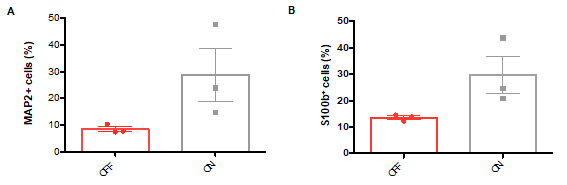


**Supplementary Figure 7:** **Expression of MAP2 and S100β in *Klover* ON conditions after seven days of differentiation.**No statistically significant increase was found in the number of mature neurons (A) and astrocytes (B) in the human hippocampal progenitor cell line HPCOA07/03 in Kl over-expressing conditions.

**Generation of Kl knocked-down cell line.**To further investigate the hypothesis that Kl is required for hippocampal neurogenesis in a controlled system, Kl was down-regulated in the human hippocampal progenitor cell line HPCOA07/03. These cells were transfected with three different Kl binding stealth RNA interference (siRNAs) or a mix of all three. The table below shows the siRNA sequences used.

| **siRNA sequences used in HPCOA07/03 against human Kl:** |
| --- |
| **CCUGAGGCAACUGCUUUCCUGGAUU**  **AAUCCAGGAAAGCAGUUGCCUCAGG Exon 2** |
| **GGACUCUUCUAUGUUGACUUUCUAA**  **UUAGAAAGUCAACAUAGAAGAGUCC Exon 3** |
| **CCCGAAAGUCUUUACUGGCUUUCAU**  **AUGAAAGCCAGUAAAGACUUUCGGG Exon 5** |

**Supplementary Table 4: Sequences of stealth siRNA (Invitrogen) that specifically binds to Kl mRNA in the cytoplasm.**

**siRNA transfection of HPCOA07/03 cells.**Following a 24-hour period in proliferating conditions, cells were transfected with three different Kl binding siRNAs (I, II or III) or a mix of all three (I-III) using the N-TER Nanoparticle siRNA Transfection System.


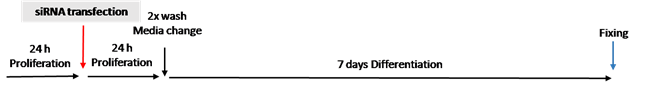


**Supplementary Figure 8: Timeline for siRNA transfection of HPCOA07/03 cells.** Cells were kept for 24 hours under proliferation condition and then transfected using the N-TER Nanoparticle siRNA Transfection System. After transfection was started, cells were maintained for seven days.

**Immunocytochemistry.**To overcome non-specific background staining of the secondary antibodies that were raised in donkey, cultures were blocked with PBS containing 5% Normal Donkey Serum (NDS) and 0.1% Triton-X-100, for 1 hour at RT and then incubated with one or two primary antibodies overnight at 4°C. The next day cultures were rinsed three times with PBS, blocked for 30 minutes at RT, and incubated with the appropriate secondary antibody for 2 hours at RT. For a nuclear counterstain, cultures were rinsed in PBS containing 300 nM 4',6- diamidino-2-phenylindole (DAPI) (Sigma) for 2 minutes followed by two PBS washes. DAPI binds strongly to DNA of fixed and live cells. Finally, cultures were either in case cells were grown on glass objective slides using the reusable chamber slides mounted with cover slips and ProLong® Gold (Invitrogen) or, in the case of 96-well plates, stored in 200 μl PBS plus 0.05% sodium azide at 4°C.

The table below contains information about the codes and dilutions of the primary and secondary antibodies used in the *in vitro* assays of the present study.

| **Primary antibodies** | **Dilution** | **Secondary antibodies** | **Dilution** |
| --- | --- | --- | --- |
| **x-Ki67: Abcam ab15580** | 1:500 | Anti-rabbit 594(Invitrogen A21207) | 1:500 |
| **x-DCX: Abcam ab18723** | 1:1000 | Anti-rabbit 488(Invitrogen A21206) | 1:500 |
| **x-MAP2: Abcam ab11267** | 1:500 | Anti-mouse 594 (Invitrogen A21203) | 1:500 |
| **x-CC3: CellSignalling #9664** | 1:500 | Anti-rabbit 594 (Invitrogen A21207) | 1:500 |
| **x-Klotho: Kyowa Hakko Kirin KM2119** | 1:500 | Anti-rat 488 (Invitrogen A21208) | 1:500 |
| **x-S100b; Dako**  **Z0311** | 1:500 | Anti-rabbit 594  (Invitrogen A21207) | 1:500 |

**Supplementary Table 5: Antibodies used for immunocytochemistry of *Klover* and Kl knocked-down HPCOA07/03 cells.**CC3= Cleaved Caspase-3; DCX= doublecortin; MAP2= microtubule-associated protein 2; S100B= S100 calcium binding protein B.

***Klotho* is required *in vivo* at different stages of AHN in a region-specific manner**

**Cell proliferation in the DG of Kl knockout mice (*kl/kl*).**Detailed methods for immunostaining of free-floating sections against Ki-67 can be found in the Materials & Methods section of this article. As shown in the figure below, no significant differences in cell proliferation were found in the DG of *kl/kl*mice (DH: 2895 ± 507.8 Wt, n=7; 2508 ± 536.0 *kl/kl*, n=5; p=0.6186; VH: 1479 ± 179.2 Wt, n=7; 1708 ± 356.7 *kl/kl*, n=5; p= 0.5453).


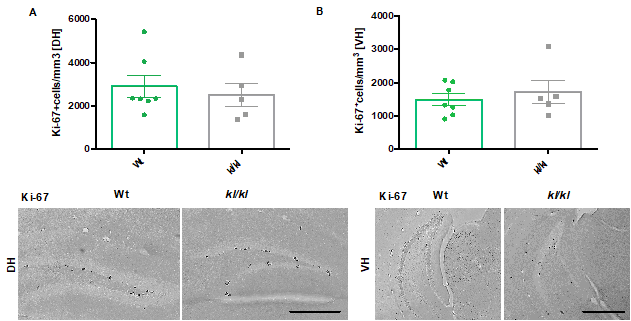


**Supplementary Figure 9. Cell proliferation in the DG of Kl knockout mice (*kl/kl*).** In alignment with *in vitro* data, no significant differences in cell proliferation were found in the DG of *kl/kl*mice, as shown by stereological quantification of Ki-67+ cells. DH = dorsal hippocampus; *kl/kl* = Klotho knockout mice; VH = ventral hippocampus; Wt = wild-type.

**Dendrite quantitative morphometric analysis of neuroblasts in the DG of Kl knockout mice (*kl/kl*).**Methods for this analysis were the same as followed for the dendrite quantitative morphometric analysis of neuroblasts in the DG of AL, CR and IF mice. As shown in the figure below, no differences in the number of branches or dendritic length were found in either the dorsal (DH) or ventral (VH) DG of *kl/kl*mice when compared with the control group (branches/DH neuroblast:8.981±0.6230 Wt, n=54; 8.483±1.067 *kl/kl,*n=29; p= 0.6668; branches/VH neuroblast: (7.818±0.5727 Wt, n=44;7.382±0.7751 *kl/kl*, n=34; p= 0.6451; length(μm)/neuroblast DH: 375.0±21.88 Wt,  n=54; 297.3±42.80 *kl/kl*, n=29; p=0.0762; length(μm)/neuroblast VH: 331.3±20.01 Wt, n=44; 302.3±34.71 *kl/kl*, n=34; p= 0.4470):


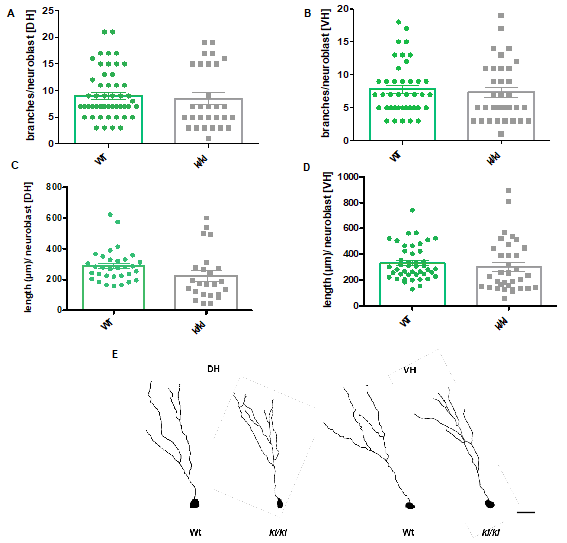


**Supplementary Figure 10: Number of branches and length of dendrites in neuroblasts in the DG of *kl/kl*and wild-type mice.**No differences were found in either parameter in either dorsal (DH) or ventral (VH) DH of *kl/kl*and wild-type (Wt) mice. DH = dorsal hippocampus; *kl/kl* = Klotho knockout mice; VH = ventral hippocampus; Wt = wild-type mice. Scale bar= 25um.

----------------------------------------------------------------------------------------------------------

Adjusted p-values for group comparisons using Tukey's multiple comparisons test following the One-Way ANOVA analyses undertaken across the study can be found below:

| **Experiment** (Diet) | **Adjusted P-value** |
| --- | --- |
| Average food intake (Fig 1A) | AL vs. CR: 0.0003  AL vs. IF: 0.0006  CR vs. IF: 0.9543 |
| MWM (probe trial 24h; Fig 1C) | AL  Zone A vs. Zone B: 0.2155  Zone A vs. Zone C: 0.9897  Zone A vs. Zone D: 0.9988  Zone B vs. Zone C: 0.1159  Zone B vs. Zone D: 0.1613  Zone C vs. Zone D: 0.9986  CR  Zone A vs. Zone B: 0.0021  Zone A vs. Zone C: 0.9962  Zone A vs. Zone D: 0.999  Zone B vs. Zone C: 0.0044  Zone B vs. Zone D: 0.0034  Zone C vs. Zone D: 0.9998  IF  Zone A vs. Zone B: 0.0463  Zone A vs. Zone C: 0.4846  Zone A vs. Zone D: 0.4846  Zone B vs. Zone C: 0.0006  Zone B vs. Zone D: <0.0001  Zone C vs. Zone D: 0.8929 |
| MWM (probe trial 10d; Fig 1D) | AL  Zone A vs. Zone B: 0.5935  Zone A vs. Zone C: 0.0512  Zone A vs. Zone D: 0.6098  Zone B vs. Zone C: 0.5317  Zone B vs. Zone D: >0.9999  Zone C vs. Zone D: 0.5155  CR  Zone A vs. Zone B: 0.8852  Zone A vs. Zone C: 0.5787  Zone A vs. Zone D: 0.7258  Zone B vs. Zone C: 0.9463  Zone B vs. Zone D: 0.2946  Zone C vs. Zone D: 0.1012  IF  Zone A vs. Zone B: 0.0614  Zone A vs. Zone C: 0.9633  Zone A vs. Zone D: 0.3314  Zone B vs. Zone C: 0.0173  Zone B vs. Zone D: 0.0003  Zone C vs. Zone D: 0.6177 |
| BrdU (24h) (Fig 2B) | AL vs. CR: 0.0085  AL vs. IF: <0.0001  CR vs. IF: 0.0390 |
| BrdU (4w) (Fig 2C) | AL vs. CR: 0.0113  AL vs. IF: 0.0072  CR vs. IF: 0.9661 |
| DCX (Fig 2E) | AL vs. CR: 0.2862  AL vs. IF: 0.0006  CR vs. IF: 0.0058 |
| %BrdU+NeuN (Fig 2F) | AL vs. CR: 0.3537  AL vs. IF: 0.0012  CR vs. IF: 0.0002 |
| Kl mRNA (fold change; Fig 3A) | AL vs. CR: 0.8968  AL vs. IF: 0.0004  CR vs. IF: 0.0005 |
| Kl (number of pixels per DG; Fig 3B) | AL vs. CR: 0.9973  AL vs. IF: 0.0425  CR vs. IF: 0.0463 |
| Kl^+^ cells per DG (Fig 3C) | AL vs. CR: 0.8656  AL vs. IF: 0.5747  CR vs. IF: 0.8252 |
| **Experiment** (*in vitro* assays) | **Adjusted P-value** |
| Kl^+^ cells (%; Fig 4E) | co siRNA vs. I: 0.0008  co siRNA vs. II: 0.0028  co siRNA vs. III: 0.0016  co siRNA vs. I-III: 0.0002  I vs. II: 0.2689  I vs. III: 0.7016  I vs. I-III: 0.1052  II vs. III: 0.8302  II vs. I-III: 0.0132  III vs. I-III: 0.0307 |
| Ki-67^+^ cells (%; Fig 4F) | co siRNA vs. I: 0.9949  co siRNA vs. II: 0.5914  co siRNA vs. III: 0.5889  co siRNA vs. I-III: 0.6364  I vs. II: 0.7983  I vs. III: 0.7961  I vs. I-III: 0.8363  II vs. III: >0.9999  II vs. I-III: >0.9999  III vs. I-III: >0.9999 |
| DCX^+^ cells (%; Fig 4G) | co siRNA vs. I: 0.9881  co siRNA vs. II: 0.0214  co siRNA vs. III: 0.4508  co siRNA vs. I-III: 0.0140  I vs. II: 0.0208  I vs. III: 0.3407  I vs. I-III: 0.0141  II vs. III: 0.2047  II vs. I-III: 0.9935  III vs. I-III: 0.1295 |
| MAP2^+^ cells (%; Fig 4H) | co siRNA vs. I: 0.9657  co siRNA vs. II: >0.9999  co siRNA vs. III: 0.0442  co siRNA vs. I-III: 0.1764  I vs. II: 0.9429  I vs. III: 0.0208  I vs. I-III: 0.0822  II vs. III: 0.0500  II vs. I-III: 0.1989  III vs. I-III: 0.8777 |
| Caspase^+^ cells (%; Fig 4I) | co siRNA vs. I: 0.0723  co siRNA vs. II: 0.0249  co siRNA vs. III: 0.0391  co siRNA vs. I-III: 0.0028  I vs. II: 0.9546  I vs. III: 0.9939  I vs. I-III: 0.2640  II vs. III: 0.9981  II vs. I-III: 0.5931  III vs. I-III: 0.4355 |
| **Experiment** (Supplementary Material) | **Adjusted P-value** |
| DCX^+^ cells/mm^3^ [DH] (diet mice; SFig 2AA) | AL vs. CR: 0.6180  AL vs. IF: 0.0153  CR vs. IF: 0.0488 |
| DCX^+^ cells/mm^3^ [VH] (diet mice; SFig 2AB) | AL vs. CR: 0.5554  AL vs. IF: 0.0164  CR vs. IF: 0.0676 |
| Total number of dendritic branches per cell (diet mice; SFig 2BA) | AL vs. CR: 0.3605  AL vs. IF: 0.9548  CR vs. IF: 0.0717 |
| Dendritic length (diet mice; SFig 2BB) | AL vs. CR: 0.5530  AL vs. IF: 0.7489  CR vs. IF: 0.0811 |
| Expression of chosen genes (diet mice; SFig 4) | Agxt2l1:  AL vs. CR: 0.0334  AL vs. IF: 0.9970  CR vs. IF: 0.0306    Ap2b1:  AL vs. CR: 0.3205  AL vs. IF: 0.0217  CR vs. IF: 0.1533  Aqp1:  AL vs. CR: 0.4099  AL vs. IF: 0.0003  CR vs. IF: 0.0008  Camk2a:  AL vs. CR: 0.0396  AL vs. IF: 0.0151  CR vs. IF: 0.6973  Cirbp:  AL vs. CR: 0.0871  AL vs. IF: 0.0346  CR vs. IF: 0.0023  Enpp2:  AL vs. CR: 0.4655  AL vs. IF: 0.0004  CR vs. IF: 0.0010  Klotho:  AL vs. CR: 0.9335  AL vs. IF: 0.0022  CR vs. IF: 0.0030  Prlr:  AL vs. CR: 0.9788  AL vs. IF: 0.2346  CR vs. IF: 0.2988  Sgk:  AL vs. CR: 0.0010  AL vs. IF: 0.9719  CR vs. IF: 0.0013  Tle1:  AL vs. CR: 0.9259  AL vs. IF: 0.7871  CR vs. IF: 0.9531 |

**Supplementary Table 6:** Adjusted p-values for group comparisons using Tukey's multiple comparisons test following the One-Way ANOVA analyses undertaken across the study.

**References:**

1. Kurosu H, Yamamoto M, Clark JD, Pastor JV, Nandi A, Gurnani P, et al. Suppression of aging in mice by the hormone Klotho. Science. 2005;309(5742):1829-33.

2. Thuret S, Toni N, Aigner S, Yeo GW, Gage FH. Hippocampus-dependent learning is associated with adult neurogenesis in MRL/MpJ mice. Hippocampus. 2009;19(7):658-69.

3. Gundersen HJ, Bagger P, Bendtsen TF, Evans SM, Korbo L, Marcussen N, et al. The new stereological tools: disector, fractionator, nucleator and point sampled intercepts and their use in pathological research and diagnosis. APMIS. 1988;96(10):857-81.

4. Paxinos G, Franklin KB. Paxinos and Franklin's the mouse brain in stereotaxic coordinates: Academic Press; 2019.

5. Plumpe T, Ehninger D, Steiner B, Klempin F, Jessberger S, Brandt M, et al. Variability of doublecortin-associated dendrite maturation in adult hippocampal neurogenesis is independent of the regulation of precursor cell proliferation. BMC Neurosci. 2006;7:77.

6. Srivastava DP, Copits BA, Xie Z, Huda R, Jones KA, Mukherji S, et al. Afadin is required for maintenance of dendritic structure and excitatory tone. J Biol Chem. 2012;287(43):35964-74.

7. Tanti A, Rainer Q, Minier F, Surget A, Belzung C. Differential environmental regulation of neurogenesis along the septo-temporal axis of the hippocampus. Neuropharmacology. 2012;63(3):374-84.

8. Irizarry RA, Bolstad BM, Collin F, Cope LM, Hobbs B, Speed TP. Summaries of Affymetrix GeneChip probe level data. Nucleic Acids Res. 2003;31(4):e15.

9. Tusher VG, Tibshirani R, Chu G. Significance analysis of microarrays applied to the ionizing radiation response. Proc Natl Acad Sci U S A. 2001;98(9):5116-21.

10. Li C, Hung Wong W. Model-based analysis of oligonucleotide arrays: model validation, design issues and standard error application. Genome Biol. 2001;2(8):RESEARCH0032.

11. Aimone JB, Gage FH. Unbiased characterization of high-density oligonucleotide microarrays using probe-level statistics. J Neurosci Methods. 2004;135(1-2):27-33.

12. Barkho BZ, Song H, Aimone JB, Smrt RD, Kuwabara T, Nakashima K, et al. Identification of astrocyte-expressed factors that modulate neural stem/progenitor cell differentiation. Stem Cells Dev. 2006;15(3):407-21.

13. Altschul SF, Madden TL, Schaffer AA, Zhang J, Zhang Z, Miller W, et al. Gapped BLAST and PSI-BLAST: a new generation of protein database search programs. Nucleic Acids Res. 1997;25(17):3389-402.

14. Geier GE, Modrich P. Recognition sequence of the dam methylase of Escherichia coli K12 and mode of cleavage of Dpn I endonuclease. J Biol Chem. 1979;254(4):1408-13.

15. Marinus MG, Morris NR. Isolation of deoxyribonucleic acid methylase mutants of Escherichia coli K-12. J Bacteriol. 1973;114(3):1143-50.
